# Spatio-Temporal Variability Description of the Rare Species *Lilium martagon* L. in Different Habitat Conditions

**DOI:** 10.3390/biology15050398

**Published:** 2026-02-28

**Authors:** Tomasz Wójcik, Kinga Kostrakiewicz-Gierałt, Maria Ziaja

**Affiliations:** 1 Department of Nature Protection and Landscape Ecology, Institute of Agricultural Sciences, Environment Management and Protection, Faculty of Technology and Life Sciences, University of Rzeszów, Zelwerowicza 4, 35-601 Rzeszów, Poland; twojcik@ur.edu.pl; 2 Department of Tourism Geography and Ecology, Institute of Tourism, Faculty of Tourism and Recreation, University of Physical Education in Kraków, Jana Pawła II 78, 31-571 Kraków, Poland; 3 Department of Tourism and Recreation, Faculty of Physical Culture Sciences, University of Rzeszów, Cicha 2A, 35-312 Rzeszów, Poland; mziaja@ur.edu.pl

**Keywords:** *Lilium martagon*, oak-hornbeam forest, mountain beech forest, developmental stages, individual traits, stems, population

## Abstract

Martagon Lily, *Lilium martagon*, belongs to geophytes inhabiting mainly forest communities in temperate regions of Europe and Asia and it is considered as a rare and endangered species in many regions. The presented investigations were conducted in three populations, occurring in forest habitats in Southern Poland: Wolski Forest (population 1), Mount Chełm (population 2), and Hrabeński Forest (population 3). At each site, 10 phytosociological relevés covering an area of 100 m^2^ were taken. For each phytosociological relevé, the Shannon–Wiener, Pielou, and Simpson indices, as well as the number of species, were calculated. The detailed field studies were conducted in permanent study patches measuring 20 m × 20 m. The measurements of habitat conditions (e.g., number of species, soil moisture, light intensity at ground level, height of plant cover) were carried out in 2018. The observations of the abundance and developmental structure of stems, as well as selected traits (e.g., height, number and dimensions of leaves, number of flowers) were conducted in 2018–2023. The analysis of phytosociological relevés showed that the study sites in Wolski Forest and Mount Chełm were located in the *Tilio cordatae–Carpinetum betuli* oak-hornbeam forest association, while the study site in Hrabeński Forest was situated in the *Dentario glandulosae–Fagetum* mountain beech forest association. The statistical analysis confirmed that the greatest Shannon and Simpson index values, number of species, soil humidity, light intensity at ground level, and height of plant cover were recorded in Hrabeński forest. The greatest number of *Lilium martagon* stems and a lack of juvenile stems was found in population 3, while in less abundant populations—1 and 2—juvenile, immature, virginile, and generative stems were found. The statistical analysis showed that the highest immature and virginile stems with the greatest number of whorl leaves, as well as the substantial height of generative stems and number of whorl leaves observed in population 3, might be the result of growing in conditions of lateral shading provided by adjacent plants. The lowest height of immature and virginile stems recorded in population 1 and generative stems noticed in population 2 might be caused by them being overshaded by the canopy of surrounding trees. Moreover, the obtained results suggest the favourable impact of weather conditions during the meteorological spring and summer of 2019 on the growth of *Lilium martagon* stems. Nevertheless, the lack of a unified trend in the studied populations indicates the occurrence of site-specific temporal variability of individual traits. Considering the obtained results, it can be concluded that population 3 presents a much better state and prospects for persistence in the occupied site than populations 1 and 2. At the same time, it should be pointed out that further long-term observations of populations of *Lilium martagon* located in different habitat conditions are still strongly desired.

## Simple Summary

The investigations of the spatio-temporal variability of the rare species *Lilium martagon* L. were conducted in three populations: population 1 (located in Wolski Forest), population 2 (in Mount Chełm), and population 3 (in Hrabeński Forest). All aforementioned localities were situated in Southern Poland. The field studies were conducted in the years 2018–2023 in permanent study patches that differed regarding habitat conditions, as well as population abundance, structure, and individual traits. The greatest number of *Lilium martagon* stems and their substantial dimensions were recorded in population 3. The lack of juvenile stems was found in population 3, while in less abundant populations—1 and 2—juvenile, immature, virginile, and generative stems were found. The investigations performed suggest the favourable impact of weather conditions during the meteorological spring and summer of 2019 on the growth of *Lilium martagon* stems. Nevertheless, the lack of a unified trend in the studied populations indicates the occurrence of site-specific temporal variability of individual traits. Considering the obtained results, it can be concluded that population 3 presents a much better state and prospects for persistence in the occupied site than populations 1 and 2.

## 1. Introduction

The genus *Lilium* consists of approximately 100 species and thousands of cultivars. These plants are found in temperate areas such as East Asia, Europe, and North America in the northern hemisphere, including 22 species in Europe. The genus is divided into seven sections, according to their bulb characteristics, leaf inflorescence, flower pattern, and origin, including *Martagon*, *Archelirion*, *Sinomartagon*, *Daurolirion*, *Leucolirion*, *Pseudolirium*, and *Liriotypus* [1,2]. *Lilium* is widely cultivated as an ornamental plant in Europe, North America, and Eastern Asia, playing a special role in garden design [3,4].

*Lilium martagon* L. (Martagon Lily) belongs to the Martagon section of the large Liliaceae family [5]. It is a 40–150 cm tall perennial with an underground golden-yellow, scaly bulb. In the central part of the stem, the leaves are arranged in characteristic whorls, usually one, sometimes two or three, while the remaining single oblong-ovate leaves, 8–12 cm long and 2–5 cm wide, are arranged spirally. It blooms from June to July. The fragrant, pendulous flowers are gathered in loose clusters at the top of the stem, numbering 3–12. The perianth tepals are 3–4 cm long, recurved upward, and pinkish-purple with darker spots. The fruit is an elongated, ribbed capsule bursting with three slits. The seeds are flat, broadly winged. It reproduces both generatively and vegetatively by producing new bulbs at the base of the mother bulb [5,6]. *Lilium martagon* is a geophyte, preferring partial shade and thermal conditions ranging from moderately warm to moderately cold. It grows in fresh, eutrophic soils with neutral and alkaline pH [7,8].

The aforementioned species has a wide range, covering temperate regions of Europe and Asia, extending from western, central, and eastern Europe to Mongolia (except Great Britain, Belgium, the Netherlands, the Scandinavian countries, and northern Russia) [9,10,11,12]. In Poland, it occurs quite often, except in the northwestern and western parts of the country [13]. Due to its wide distribution range, *Lilium martagon* grows in numerous plant communities. It is most frequently recorded in eutrophic and mesotrophic deciduous forests. In Poland, it is widespread in the Carpathians and Sudetes, from the foothills to the subalpine zone [14,15,16,17,18,19,20,21,22], and is less common in the lowlands [23,24]. It is a characteristic species of meso- and eutrophic deciduous forests of the order *Fagetalia sylvaticae* and a distinctive species of the *Bupleuro–Calamagrostietum arundinaceae* association, a group of high-mountain herbaceous plants from the *Calamagrostion* alliance [25]. Moreover, it also occurs in beech forests in the Eastern Alps, the Bohemian Massif and the Western Carpathians [26], subalpine beech forests in the Julian Alps, and in the northern Dinaric Alps [27], as well as in southeastern Europe in the Balkan Peninsula, from the southeastern areas of the Alps in Slovenia, through Croatia, Bosnia and Herzegovina, Serbia, Montenegro, and the Republic of Macedonia to northern and northeastern Greece and Bulgaria (alliance *Aremonio-Fagion* and *Fagion moesiacae*) [28]. Furthermore *Lilium martagon* occurs in oak-hornbeam forests, including the subcontinental oak-hornbeam forest *Tilio cordatae–Carpinetum betuli* [14,21,29] and the Central European *Galio silvatici–Carpinetum* [23], and is recorded in riparian forests of the *Alno–Ulmion* alliance [19,30,31] and in the subcontinental oak-hornbeam forest association *Potentillo albae–Quercetum* [32]. Additionally, it was noted in Carpathian mountain grasslands in the associations *Festucetum saxatilis*, *Ranunculo platanifolii–Adenostyletum alliariae*, *Hyperico alpigeni–Calamagrostietum villosae* [33,34], and Alpine pastures [35]. This species can also be found sporadically in anthropogenic habitats, such as old parks and cemeteries [36,37].

In Poland, *Lilium martagon* is under strict species protection [38] and classified in various threat categories depending on the region. It is considered as an endangered species (EN) in the Suwałki Lakeland [39], a vulnerable species (VU) in Western Pomerania [40] and the South Podlasie Lowland [41], whilst a near threatened species (NT) in Gdańsk Pomerania [42] and in the Silesian Voivodeship [43]. Moreover, Martagon Lily is considered as a species of least concern (LC) in Lower Silesia [44], in the Opole Voivodeship [45], and in Greater Poland [46], and as a low-risk species (LR) in the Proszowice Plateau [47]. It is also listed as endangered in neighbouring countries. *Lilium martagon* is included in the Red List of Vascular Plants of the Czech Republic with the NT category [48], the Red List of Vascular Plants of the Ukrainian flora with the LC category [49], the Red List of Vascular Plants of the Carpathians with the LC category [50], and the Red Book of Vascular Flora of Croatia with the VU category [51]. It has also been included in the European Red List of Medicinal Plants [52].

To date, the majority of investigations have focused on taxonomic and phylogenetic relationships among particular taxa representing the genus *Lilium* [11,14] or have addressed the issue of reproduction or development of individuals [53,54,55,56,57,58,59].

Due to the threat of extinction of *Lilium martagon*, studies of its population abundance and structure, as well as observations of individual traits, are particularly important because they allow for an evaluation of the prospects for persistence of the population in the occupied habitat and are the basis for undertaking specific protective actions. So far, such studies have been conducted among others in Poland [23,24,60,61,62,63,64,65,66], Ukraine [67], Russia [68], and Latvia [10,69]. Nevertheless, the aforementioned studies addressed solely selected individual traits and were conducted over one [16,23,29,60,65], two [62,66], or three [10] growing seasons in a low number of study sites. Longer observations lasting at least four years were carried out by Balode [69] and Pindel [63]. The longest six-year investigations of individual traits of *Lilium martagon* were carried out by Miciniak and Zątek [24]. However, the aforementioned research was conducted in selected years of a six-year period. All these authors analysed the influence of habitat conditions, such as light intensity and soil conditions as well as floristic and phytocoenotic variability of ecosystems on the state of *Lilium martagon*. The influence of weather conditions on Martagon Lily population was investigated exclusively by Truchan and Sobisz [64]. Based on three-year observations conducted at an anthropogenic locality, they confirmed the negative effect of precipitation on population abundance. Despite growing interest in the aforementioned issues, the present state of knowledge is still insufficient. Therefore, the presented six-year study was undertaken to evaluate the spatial and temporal variability of population and individual traits of *Lilium martagon*. The specific aims focused on assessing: (i) habitat conditions at all study sites, (ii) weather conditions in the study period, (iii) abundance and structure of the studied populations of *Lilium martagon*, and (iv) selected traits of stems of *Lilium martagon* representing different developmental stages.

## 2. Materials and Methods

### 2.1. Study Area

The investigations were conducted in three *Lilium martagon* localities in Southern Poland. Site 1 is located in Wolski Forest in the western part of Kraków (50.057617° N, 19.885717° E), within the Bielańsko-Tyniecki Landscape Park. In terms of physico-geographical regionalisation, this area is located in the Kraków Bridge mesoregion of the Kraków Gate macroregion [70]. A characteristic feature of this area is its varied relief, within which various landforms can be distinguished. The highest parts of the hills are occupied by a plateau, covered with loess deposits, on which a *Pino–Quercetum* mixed coniferous forest has developed. The slopes are cut by deep valleys; brown soils and rendzinas occurring here are covered by *Tilio cordatae–Carpinetum betuli* and *Dentario glandulosae–Fagetum* associations. On limestone rocks and in their surroundings, xerothermic grasslands representing the *Festuco–Brometea* class and shrubs of the *Rhamno–Prunetea* class can be found [71,72]. The observed *Lilium martagon* population (called in this study population 1) occurs in the *Tilio cordatae–Carpinetum betuli* association at an altitude of 243 m above sea level in the eastern part of Wolski Forest.

Site 2 is located on Mount Chełm in the village of Jaszczurowa (49.888957° N, 21.559002° E). This area is situated within the Mount Chełm Nature Reserve and the Czarnorzecko-Strzyżowski Landscape Park. In terms of physico-geographical regionalisation, site 2 is located in the Strzyżów Foothills mesoregion, in the Mid-Beskidy Foothills macroregion [70]. Mount Chełm is composed of Carpathian flysch, with thick-bedded sandstones in the substrate. The aforementioned reserve protects an extrazonal site of the *Dentario glandulosae–Fagetum* association, an area of springs with the *Carici remotae–Fraxinetum* association, the *Tilio cordatae–Carpinetum betuli* association, and an abandoned quarry [73,74]. The population of *Lilium martagon* (labelled population 2) occurs in the top part of the Mount Chełm (520 m above sea level) in a *Dentario glandulosae–Fagetum* association.

Site 3 is located in Hrabeński Forest in the village of Besko (49.594234° N, 21.920799° E), situated in the Jasło–Krosno Basin mesoregion, in the Mid-Beskidy Foothills macroregion [70] and is protected within the Natura 2000 Hrabeński Forest PLH180039 site. The substrate consists of Carpathian flysch composed of a series of alternating shales and sandstones. Hrabeński Forest occupies the hills between the Wisłok and Tabor river valleys, where the elevation differences reach up to 100 m. In the lowest part of the forest, on gentle slopes, the *Tilio cordatae–Carpinetum betuli* association is found. In the higher parts of the hills, on slopes with greater inclination and varied exposure, Carpathian beech forest occurs in the rank of two subassociations: *Dentario glandulosae–Fagetum typicum* and *Dentario glandulosae–Fagetum lunarietosum* [21]. The population of *Lilium martagon* (called population 3) occurs throughout the whole forest complex; however, it is most abundant in the *Tilio cordatae–Carpinetum betuli* association at an altitude of 255 m above sea level.

### 2.2. Data Collection

In 2018, the investigations of plant cover and habitat conditions were conducted at three study sites: Wolski Forest, Mount Chełm, and Hrabeński Forest. At each site, 10 phytosociological relevés covering an area of 100 m2 were taken using the Braun-Blanquet method [75]. The names of vascular plants are given according to Mirek et al [76]. The description of vegetation includes species covering more than 5% of a given plant layer. Syntaxonomic classification was based on the Matuszkiewicz system [25]. For each phytosociological relevé, the Shannon–Wiener diversity index [77], Pielou index [78], Simpson index [79], and number of species were calculated using JUICE 7.1 software [80]. Then, mean values of diversity indices for plant communities occurring in the three study sites were calculated.

Subsequently, within each study site one permanent research patch measuring 20 m × 20 m was established. Altogether, in each permanent patch, 10 measurements of soil pH, 20 measurements of soil moisture (%), and herbaceous vegetation height (cm), as well as 30 measurements of light intensity (lx) were taken. Soil pH was measured with a soil acidimeter (RIM KOWALCZYK SP.J., Janki, Poland) using Hellig’s solution, soil moisture with an Extech MO750 hygrometer (Extech Instruments Corporation, Maastricht, Netherlands), and light intensity at ground level with a TES1335 luxmeter (TES Electrical Electronic Corp., Taipei, Taiwan). The aforementioned measurements were conducted from 10 to 15 June 2018, from 12:00 to 15:00 in full sunlight. Additional analyses of habitat conditions were conducted using Ellenberg indicators [81]. Ellenberg indicators were calculated for each phytosociological relevé in JUICE programme [80] and then mean values for the plant communities occurring in particular study sites were computed.

In order to describe the temporal variability of weather conditions in the study period, monthly sum of precipitation [mm] and monthly mean temperature [°C] data from Kraków and Krosno stations (situated near the Mount Chełm and Hrabeński Forest sites) were taken from the website TuTiempo (Accessed on 21 March 2025) [82].

The investigations of populations of *Lilium martagon* were conducted in the years 2018–2023 within the aforementioned permanent plots measuring 20 m × 20 m. In each study year, all stems of *Lilium martagon* occurring within the permanent patches were counted. Taking into account the fact that the accurate determination of age of individuals in clonal, strictly protected populations of *Lilium martagon* in Poland [38] is possible only on the basis of bulb morphology, instead of age, the developmental stage of the stems was determined on the basis of the number of leaves [29,61,62]. Considering this, each stem was assigned to one of the following stages: (i) juvenile stage (stem with 1–2 leaves), (ii) immature stage (3–12 leaves), (iii) virginile (mature vegetative) stage (more than 12 leaves and lack of inflorescence), or (iv) generative stage (with developed inflorescence).

Next, observations of selected individual traits of the stems of *Lilium martagon* were carried out. The height, number of leaves in the whorl, and length and width of the longest leaf in the whorl were observed in immature stems. The height, number of leaves in the lower whorl, and length and width of the longest leaf in this whorl were observed in virginile stems. The height, number of leaves in the lowest whorl, length and width of the longest leaf in this whorl, number of spiral leaves, and length and width of the spiral leaf placed above the highest whorl, as well as the number of flowers, were recorded in generative stems. When the number of stems representing a particular stage reached no more than 30, all stems were measured; meanwhile, if they occurred more abundantly (at least 31 stems), the number of examined stems was limited to 30. The stems were randomly selected based on 30 throws with an iron rim, 20 cm in diameter. Each time, one stem occurring in the centre of the rim was measured and labelled.

### 2.3. Statistical Analysis

The non-parametric Kruskal–Wallis H test for multiple comparisons was applied to check the statistical significance of differences in values of:

- Shannon index, Simpson index, Pielou index, number of species, soil moisture, soil pH, height of plant cover and light intensity at ground level, as well as values of Ellenberg indices among study sites;
- Individual traits of immature stems of *Lilium martagon* (height, number of leaves in the whorl, and dimensions of the longest one) among populations in the years 2018–2023;
- Individual traits of virginile stems (height, number of leaves in the lower whorl, and dimensions of the longest one) among populations in the years 2020–2023;
- Individual traits of generative stems (height, number of flowers, number of whorls, number of leaves in the lowest whorl and dimensions of the longest one, number of spiral leaves, and dimensions of spiral leaf placed above the highest whorl) among populations in the years 2018–2023.

The non-parametric Mann–Whitney U test was used to check the statistical significance of differences in values of height, number of leaves in the lower whorl and dimensions of the longest leaf in virginile stems among populations in the years 2018 and 2019. Moreover, the non-parametric Kruskal–Wallis H test for multiple comparisons was applied to check the statistical significance of differences in values of individual traits of stems in the study years. Additionally, the d-Cohen coefficient was calculated to check the scale of practical significance of differences among pairs of data referring to habitat conditions at the study sites, as well as individual traits of stems representing different populations. The scale of practical significance of differences based on absolute values of the d-Cohen coefficient was as follows: <0.2—negligible, 0.21–0.49—small, 0.5–0.79—moderate, and >0.8—substantial difference. The χ^2^ test was used to test the statistical significance of spatial and temporal differences in the share of stems at various developmental stages. The analyses were conducted using Statistica 13.3 software and an interactive χ^2^ test calculator [83].

## 3. Results

### 3.1. Habitat Conditions

**Figure 1:**
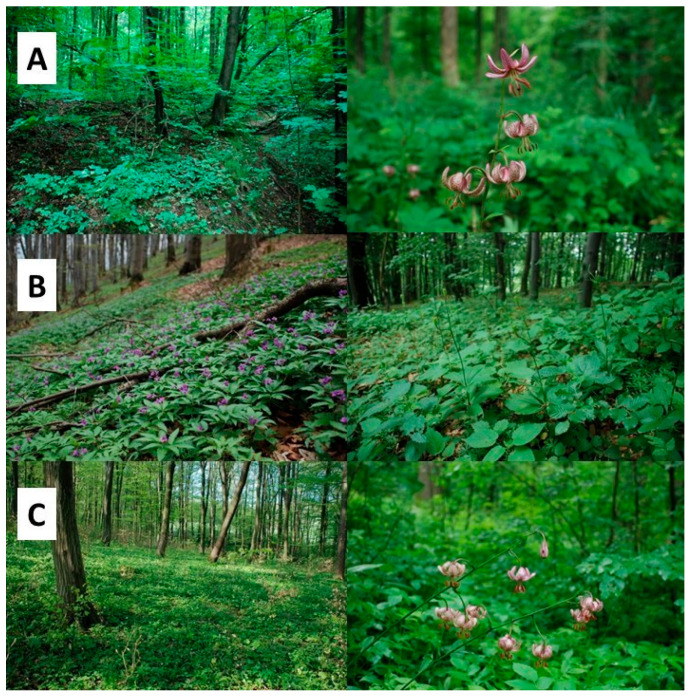
Plant communities with *Lilium martagon* stems at the study sites: (**A**)—site 1 (Wolski Forest), (**B**)—site 2 (Mount Chełm), and (**C**)—site 3 (Hrabeński Forest).

**Figure 2:**
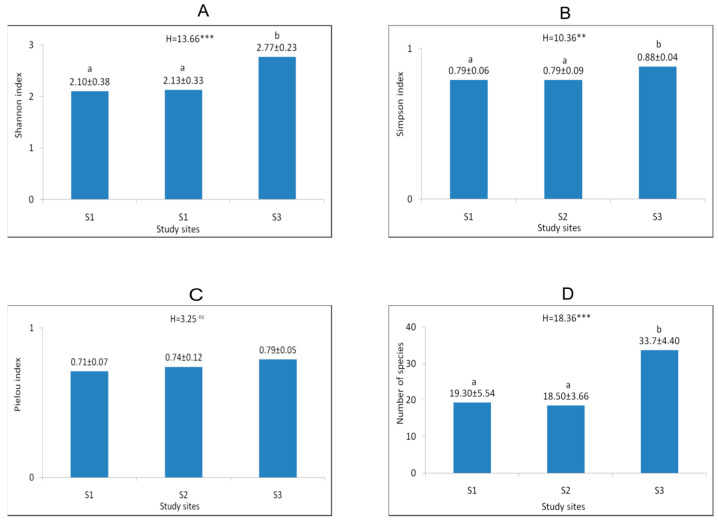
Mean (±SD) values of Shannon index (**A**), Simpson index (**B**), Pielou index (**C**), and number of species (**D**) at study site 1 (Wolski Forest), study site 2 (Mount Chełm), and study site 3 (Hrabeński Forest). The asterisks indicate statistical significance level: * *p* ≤ 0.05, ** *p* < 0.01, *** *p* < 0.001, ns-not significant (Kruskal–Wallis H test). The different letters below H values indicate significant differences among populations.

Analysis of phytosociological relevés showed that the studied sites differed in terms of vegetation cover. At sites 1 (Wolski Forest) and 3 (Hrabeński Forest), the *Tilio cordatae–Carpinetum betuli* oak-hornbeam forest association was observed, while at site 2 (Mount Chełm) the *Dentario glandulosae–Fagetum* mountain beech forest association was recorded (Table 1, Figure 1). At all sites, the tree layer was well developed, reaching an average cover of 77 to 88%. At site 1, the tree layer was dominated by *Tilia cordata* and *Quercus robur*, at site 2 *Fagus sylvatica* and *Acer pseudoplatanus* prevailed, while at site 3 *Carpinus betulus*, *Acer pseudoplatanus*, and *Quercus robur* were represented most abundantly. The shrub layer was relatively well developed in the *Tilio cordatae–Carpinetum betuli* association, where its average cover was 34% (site 3) and 45% (site 1). At site 2, the shrub layer was poorly developed, with an average cover of only 5%. The herbaceous vegetation layer achieved high, but varied, coverage across sites. It was most developed at site 3 (91% coverage), where as many as eight species achieved coverage exceeding 5%, including *Lilium martagon* and *Stellaria holostea*, a characteristic species of the oak-hornbeam forest. At site 2, the herbaceous vegetation was dominated by *Dentaria glandulosa*, a characteristic species of the *Dentario glandulosae–Fagetum* association, but *Galeobdolon luteum* and *Rubus hirtus* also had significant cover. Herbaceous vegetation at site 1 was slightly less developed (68%). Five species achieved high coverage in this layer, including *Stellaria holostea*, a species of the oak-hornbeam forest. The highest species richness was observed at site 3, where an average of 34 species per phytosociological relevé was recorded. This site also had the highest values of the Shannon–Wiener, Simpson, and Evenness indices (Figure 2), as confirmed by the values of the d-Cohen coefficient (Table A1).

**Figure 3:**
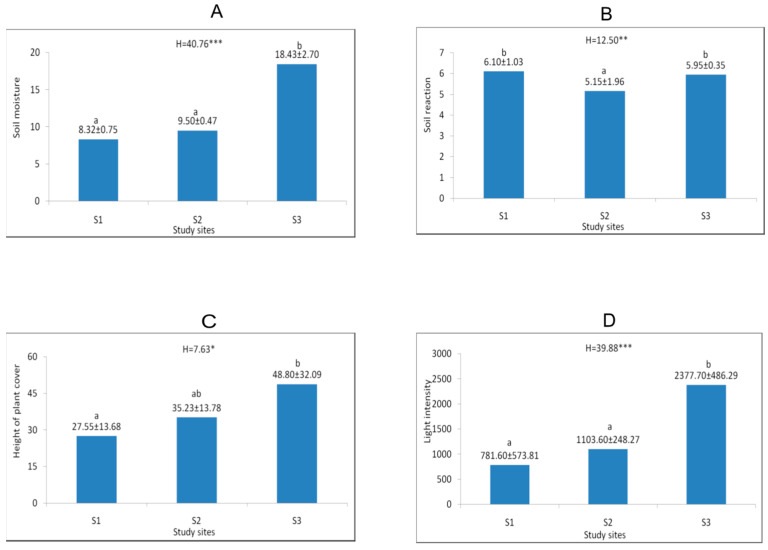
Mean (±SD) values of soil moisture (%) (**A**), soil pH (**B**), plant cover height (cm) (**C**), and light intensity at ground level (lx) (**D**) at the study sites. The explanations of abbreviations S1–S3 and description of statistical significance levels—as in Figure 2.

**Figure 4:**
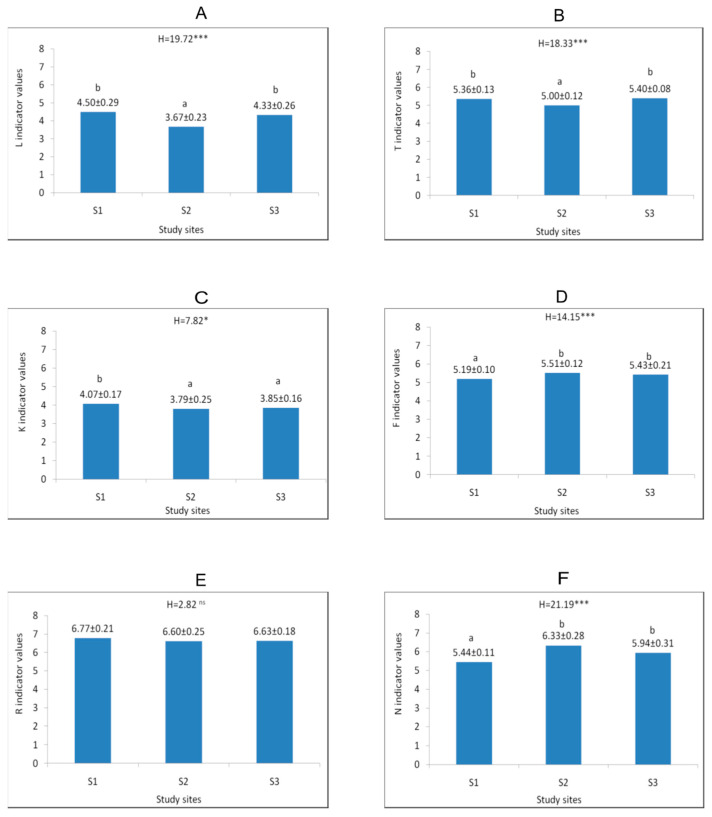
Mean (±SD) values of Ellenberg indicators of light (**A**), temperature (**B**), continentalism (**C**), moisture (**D**), soil reaction (**E**), and nitrogen content (**F**) at the study sites. The explanations of abbreviations S1–S3 and description of statistical significance levels—as in Figure 2.

The study sites differed in terms of habitat conditions. The highest values of soil moisture, light intensity at ground level, as well as height of plant cover were noticed at site 3. The values of the d-Cohen coefficient confirmed the substantial differences, especially in the case of soil moisture and light intensity (Table A1). At sites 1 and 3, located in the oak-hornbeam forests, the soil pH was neutral, while at site 2, situated in a beech forest, it was slightly acidic (Figure 3). The substantial differences in soil reaction between the oak-hornbeam and beech forests were confirmed by the d-Cohen coefficient values (Table A1). Analysis of Ellenberg’s climatic indicators revealed significantly higher L and T values at sites 1 and 3 than at site 2. In the case of edaphic indicators, significant differences were observed for F and N. They reached much greater values at sites 2 and 3 than at site 1. Additionally, the K indicator was greater at site 1 than at the sites 2 and 3 (Figure 4). All aforementioned differences were confirmed by the values of the d-Cohen coefficient (Table A2).

### 3.2. The Selected Weather Conditions in the Study Period

**Figure 5:**
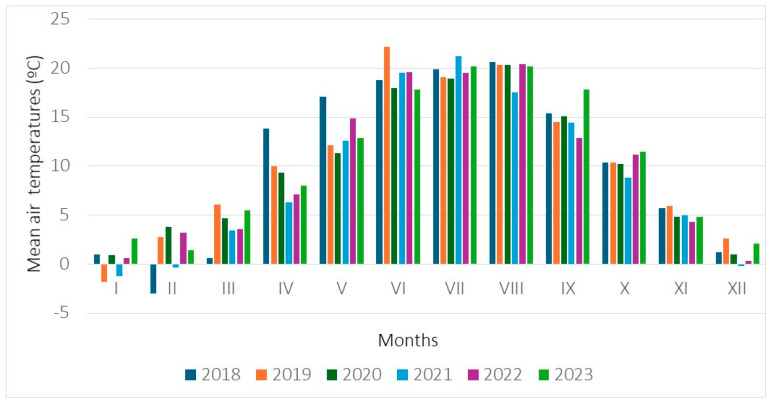
Mean monthly temperatures (°C) measured in 2018–2023 at a meteorological station located in Kraków.

**Figure 6:**
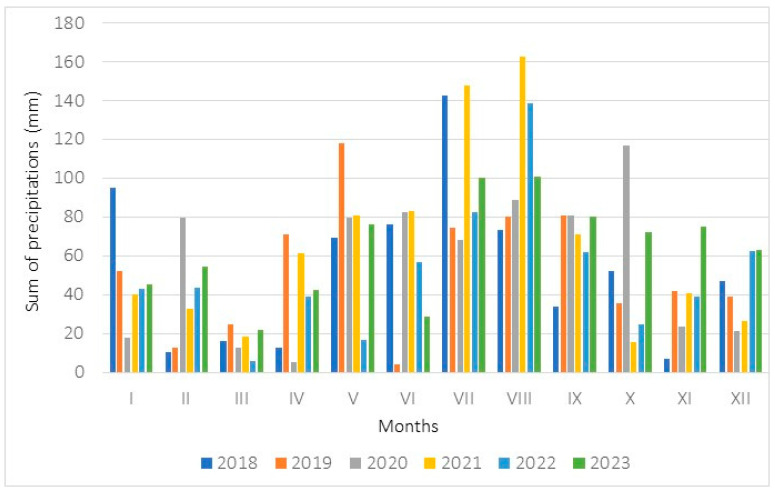
Monthly sums of precipitation (mm) measured in 2018–2023 at a meteorological station located in Kraków.

Weather conditions in the study site where population 1 occurred were evaluated on the basis of data from the Kraków station, where measurements were taken (Figure 5 and Figure 6). The annual sum of precipitation ranged from 615.1 mm (in the year 2022) to 781.5 mm (2021). The mean annual air temperatures achieved values ranging from 8.9 °C (in the year 2021) to 10.4 °C (2019, 2023). During the meteorological spring occurring from 1 March till 31 May, the sum of precipitation ranged from 61.5 mm (in the year 2022) to 214.1 mm (2019), while the mean air temperatures ranged from 7.4 °C (in the year 2021) to 10.5 °C (2018). During the meteorological summer (from 1 June to 31 August), the sum of precipitation rose from 159.3 mm (2019) to 394.0 mm (2021), while the air temperatures achieved values ranging from 19.1 °C (2020) to 20.5 °C (2019).

**Figure 7:**
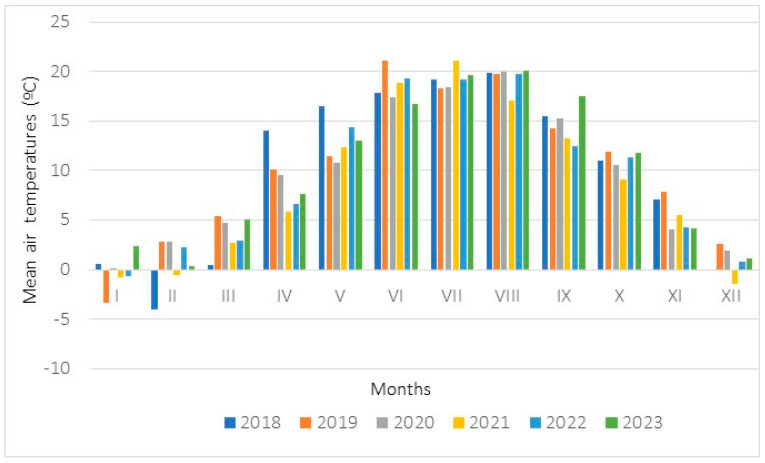
Mean monthly temperatures (°C) measured in 2018–2023 at a meteorological station located in Krosno.

**Figure 8:**
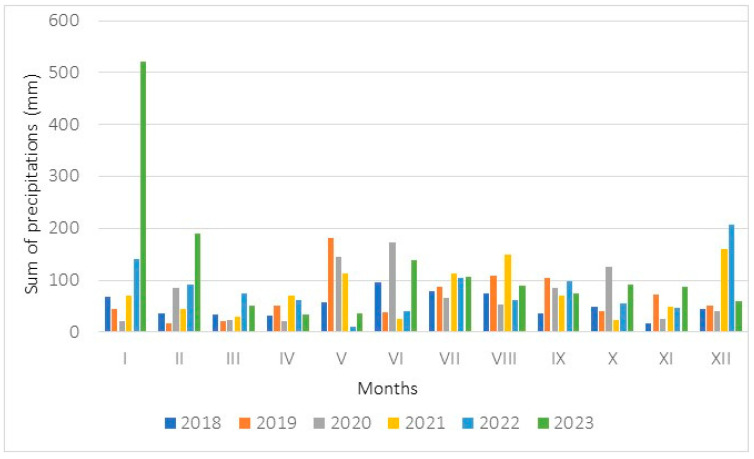
Monthly sums of precipitation (mm) measured in 2018–2023 at a meteorological station located in Krosno.

Weather conditions at the study sites where populations 2 and 3 occurred were evaluated on the basis of data from the Krosno station, where measurements were taken (Figure 7 and Figure 8). The annual sum of precipitation rose from 623.6 mm (in the year 2018) to 1483.6 mm (2023). The mean annual air temperatures achieved values ranging from 8.7 °C (2020) to 12.0 (2018). During the meteorological spring, the sum of precipitation ranged from 123.0 mm (2023) to 254.5 mm (2019), while the mean air temperatures ranged from 6.4 °C (2023) to 9.0 °C (2019). During the meteorological summer, the sum of precipitation rose from 207.5 mm (2022) to 334.7 mm (2023), while the measured mean air temperatures ranged from 17.9 °C (2020) to 19.7 °C (2019).

### 3.3. Spatio-Temporal Variability of Abundance and Structure of Developmental Stages of Populations

**Figure 9:**
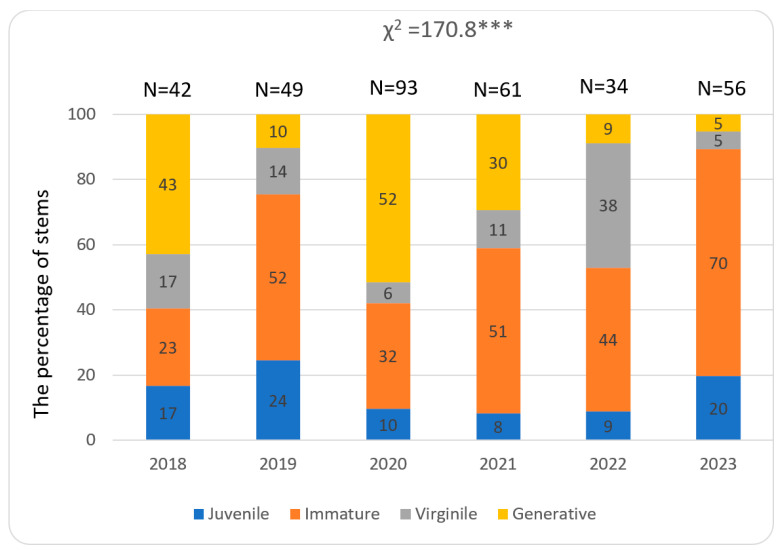
Percentage of juvenile, immature, virginile, and generative stems in population 1 located in Wolski Forest. The asterisks indicate statistical significance levels: ** *p* < 0.01, *** *p* < 0.001 (χ^2^ test).

**Figure 10:**
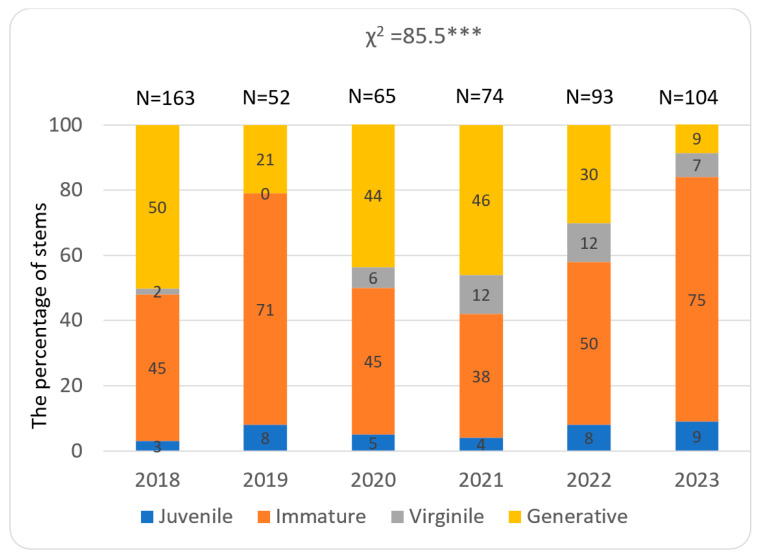
Percentage of immature, virginile, and generative stems in population 2 located on Mount Chełm. Statistical significance levels—as in Figure 9.

**Figure 11:**
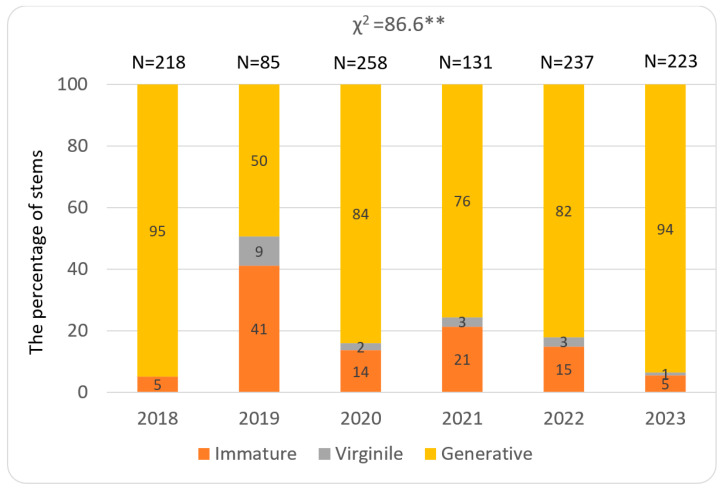
Percentage of immature, virginile, and generative stems in population 3 located in Hrabeński Forest. Statistical significance levels—as in Figure 9.

The abundance of stems of *Lilium martagon* in the study patches and structure of their developmental stages showed both spatial and temporal. The total number of stems was the greatest in population 3, where it ranged from 85 to 237 in particular study years. A much lower number of stems, ranging from 52 to 163, was recorded in population 2, while in population 1 34 to 93 stems were noted (Figure 9, Figure 10 and Figure 11).

During the whole study period in populations 1 and 2, juvenile, immature, virginile, and generative stems occurred. In population 1, the greatest share of juvenile stems (comparing other populations) especially in the year 2019 was observed, while the contribution of stems representing the remaining stages differ in particular years (Figure 9). In population 2, a share of immature stems greater than at other study sites was noted. Also, the percentage of generative stems was substantial during almost all study years (Figure 10). In population 3, juvenile stems were absent and the contribution of generative stems was much greater than in other populations (Figure 11). The temporal variability among the populations was statistically significant and ranged from χ^2^ = 47.00 (*p* < 0.001) in 2021 to 266.4 (*p* < 0.001) in 2023.

### 3.4. The Spatial Variability of Selected Individual Traits of Lilium martagon

The mean height of immature stems increased from population 1, where it achieved from 11.02 cm to 17.56 cm, via population 2 (11.53–17.12 cm), to population 3 (13.71–18.77 cm). The mean number of leaves in the whorl ranged from 5.58 to 9.40 in population 1, from 6.63 to 7.85 in population 2, and from 7.30 to 8.95 in population 3. Such phenomena were confirmed by the values of the Kruskal–Wallis H test for multiple comparisons (Table 2) and the d-Cohen coefficient (Table A3). The mean length of the longest leaf exhibited the lowest values (94.15–119.14 mm) in population 3 and was the greatest (97.90–139.28 mm) in population 1. Also, the mean width of the aforementioned leaf was the lowest (32.96–38.77 mm) in population 3 and the greatest (35.70–39.75 mm) in population 1 (Table 2). These tendencies were also confirmed by the statistical analysis (Table 2 and Table A3).

The mean height of the virginile stem was the lowest (13.14–22.86 cm) in population 1 and the greatest (22.17–31.25 cm) in population 3. The mean number of leaves in the lower whorl was the lowest (5.33–7.80) in population 1 and the greatest (8.29 to 11.00) in population 3. The differences among the populations were confirmed by small, moderate, or substantial values of the d-Cohen coefficient (Table A4). The mean length of the longest leaf ranged from 103.43 mm to 128.71 mm, while the mean width of this leaf varied from 29.40 mm to 45.57 mm, with limited variation among the populations confirmed by the Kruskal–Wallis H test (Table 3). Interestingly, the substantial values of the d-Cohen coefficient indicated a remarkable size effect in some study years (Table A4).

The mean height of generative stems achieved the lowest values (55.27–72.93 cm) in population 2 and was the greatest (58.67–97.00 cm) in population 1 (Table 4). The significant differences between populations 1 and 2 were confirmed by the d-Cohen coefficient values (Table A5). The mean number of flowers ranged from 2.17 to 2.68 in population 2, from 3.03 to 4.58 in population 3, and from 1.67 to 6.20 in population 1. The mean number of whorls reached the lowest values (1.40–1.82) in population 2, whereas the greatest number (1.44–3.00) was recorded in population 1. In the case of the number of flowers and whorls, the statistical analysis confirmed the moderate level of the d-Cohen coefficient between populations 1 and 2 (Table A5). The mean number of leaves in the lowest whorl had the lowest values in population 1 (5.57–7.00) and the greatest values in population 3 (8.00–9.47). This phenomenon was confirmed by the substantial values of the Kruskal–Wallis H test (Table 5) and the d-Cohen coefficient (Table A6).

The mean length of the longest leaf in the lowest whorl was the lowest (109.67–126.13 mm) in population 3 and the greatest (119.57–147.80 mm) in population 1. The mean width of the longest leaf achieved the lowest values (35.03–39.63 cm) in population 3 but was the greatest (34.93–43.00) in population 2. The mean number of spiral leaves ranged from 3.73 to 5.20 in population 2, from 4.30 to 5.26 in population 3, and from 4.00 to 8.50 in population 1. The values of the Kruskal–Wallis H test (Table 5) and the d-Cohen coefficient (Table A6) indicated small or moderate differences in the aforementioned leaf dimensions among the populations.

The mean number of spiral leaves reached the lowest values (3.59–5.20) in population 2 but was the greatest (4.00–8.50) in population 1 (Table 5). This phenomenon was confirmed by the values of the Kruskal–Wallis H test (Table 5) and the d-Cohen coefficient (Table A6).

The mean length of the spiral leaf above the highest whorl achieved the lowest values (81.83–94.68 mm) in population 3 and was the greatest (97.67–113.33 mm) in population 1. The mean width of the aforementioned leaf exhibited a similar tendency, ranging from 16.73 mm to 23.68 mm in population 3, from 21.10 mm to 25.13 mm in population 2, and from 20.20 mm to 30.53 mm in population 1. The greatest leaf dimensions observed in population 1 were confirmed by the values of the Kruskal–Wallis H test (Table 5) and the d-Cohen coefficient (Table A6).

### 3.5. The Temporal Variability of Selected Individual Traits of Lilium martagon

The height of immature stems in population 1 presented significantly lower values in the years 2018–2020 than in subsequent years, while in population 2 and in population 3 the highest stems were noted in the year 2019 (Figure S1, Table S1). The number of leaves in the whorl was significantly greater in the year 2019 than in other study seasons solely in population 1, while in the remaining populations this trait did not show temporal variability. The length of the longest leaf was significantly greater in the year 2019 than in other study seasons solely in population 1. In population 2, the length was lower in the years 2018–2020 than in the years 2021–2023, whereas in population 3 much lower values were observed in the years 2020, 2021, and 2023 than in other study seasons. The width of the longest leaf in population 1 was much lower in the years 2020 and 2022 than in other years; in population 2, it was much lower in the years 2018–2020 than in subsequent study seasons, while in population 3 the aforementioned trait did not present temporal variability.

The individual traits of virginile stems did not present temporal variability in population 2 or in population 3. In population 1, the height of virginile stems and the length of the longest leaf in the lower whorl reached the greatest values in the year 2021 and the lowest values in the year 2018 (Figure S2). The moderate and substantial values of the d-Cohen coefficient indicate remarkable differences in the height of stems and leaf dimensions, particularly in population 1 (Table S2).

The height of generative stems in population 1 achieved much greater values in the year 2019 than in remaining study seasons (Figure S3). In population 2, the greatest values of the aforementioned trait were observed in the years 2022, while in population 3 this trait reached its greatest values in the years 2022 and 2023. The number of flowers and whorls showed temporal variability in population 1, where this trait reached its greatest values in the year 2019, as well as in population 3, where it achieved the greatest values in the years 2022 and 2023. The substantial values of the d-Cohen coefficient regarding the height, number of flowers, and number of generative whorls indicate significant differences between 2019 and the other years (Table S3).

The number of leaves in the lowest whorl presented temporal variability solely in population 3, where it achieved the greatest values in the year 2019. This phenomenon was confirmed by the values of the Kruskal–Wallis H test (Figure S4) and the d-Cohen coefficient (Table S4). The length of the longest leaf in the lowest whorl showed temporal variability in population 2 and in population 3. In population 2, it reached the greatest values in the years 2022 and 2023, while in population 3 the greatest values were observed in the year 2019. This phenomenon was confirmed by the small and moderate values of the d-Cohen coefficient (Table S4). The width of the aforementioned leaf in population 2 was significantly greater in the years 2021 and 2022 than in other study seasons, whereas in other populations the values of this trait were similar in individual years. The number of spiral leaves showed temporal variability in population 1 and in population 2 (Figure S5, Table S4). The lowest values were observed in the year 2023 in the case of population 1 and in the year 2020 in the case of population 2. The length and width of the spiral leaf did not exhibit temporal variability.

## 4. Discussion

### 4.1. Habitat Conditions

*Lilium martagon* occurs at various geographical latitudes, both in the warmer regions of southern and western Europe and in central Asia, where the climate is distinctly continental [9,11], indicating that it is a species neutral to continentality with a wide range of climatic tolerance. In cooler regions, it prefers partially wooded ecosystems [68,84], whereas in sunnier and drier areas, it finds optimal growth conditions in dense and shaded forests [14,27,28]. The investigated populations are located within forest communities with diverse light conditions. In Hrabeński Forest, light intensity measured at the ground was more than twice as high as at the other sites. This was the most numerous population, characterised by a high proportion of flowering and fruiting individuals in all years of the study. Urbaniec and Bach [16] and Pindel [63] reported the positive effect of light on the development of *Lilium martagon*, finding that optimal conditions for the development of this species occur at light levels approaching 60%. In turn, according to Jańczyk-Węglarska and Węglarski [61], the optimal conditions for the occurrence of this species range from 5 to 20% of light reaching the herbaceous vegetation layer. According to Pindel [63], increasing stand density and reduced light access negatively impact the flowering process, and at light levels below 5% the process is completely inhibited. We observed a similar relationship at the other two sites, where lower light intensity may have negatively impacted the number of flowering individuals.

According to the literature on the subject, *Lilium martagon* can occur on various soil types (including podzolic soils, brown soils, leached brown soils, gley soils, and rendzinas) and on various formations (including loess formations and calcareous clay soils), but their common feature is a high humus content and high or moderate nitrogen content [10,16,23,61,69,85]. Such findings are consistent with the results of the presented investigations, particularly Ellenberg N index values suggesting soils moderately rich in nitrogen. However, the content of available compounds can reach diverse values. Moreover, *Lilium martagon* has a wide amplitude in relation to soil pH and it occurs in habitats ranging from very acidic to alkaline. However, many authors emphasise that the optimal pH for this species ranges from 5 to 7 [16,23,58,60,61,62,63]. The aforementioned findings are supported by the performed investigations showing that average pH value of the study sites ranged from 5.15 to 6.10.

Analysis of moisture conditions showed twice as high humidity in Hrabeński Forest, whose habitat can be described as moderately moist. At the other two sites, the soils were fresh, and during prolonged periods without rainfall in the summer, they dried out. Some authors have pointed out that individuals of *Lilium martagon* develop best in fresh habitats [7,8], but other literature data indicate that it can also occur in seasonally moist habitats, such as riparian forests [19,30,31,69]. At the same time, it is worth mentioning that excessive humidity in spring, resulting from high rainfall totals and the formation of ponds, can lead to bulb rot and the decline of entire populations [63]. A similar relationship in this type of habitat was also observed in the case of the *Arum alpinum* species [73,86].

In the temperate climate zone, individuals of *Lilium martagon* have optimal conditions for development in fertile and mesotrophic deciduous forests; therefore, this species is recognised as a characteristic species of the order *Fagetalia* [25,27]. In the lowlands and foothills, it occurs mainly in oak-hornbeam forests, including in the *Tilio cordatae–Carpinetum betuli* and *Galio sylvatici–Carpinetum betuli* associations [14,23,29,60,85,87], while it grows much less frequently in pine forests and mixed forests [69,78,88], light oak forests, [24,32] and riparian forests [19,30,31]. In the montane zone and extrazonally in the foothills, it is a frequent component of beech forests, especially the *Dentario glandulosae–Fagetum* association [14,15,16,26,27,62,85], while in higher locations it occurs in alpine grassland communities [16,33,34]. Interestingly, this species can establish itself in anthropogenic habitats, such as manor parks and cemeteries, where it was intentionally introduced [10,36,37,69]. Our study results confirm the literature data regarding the preferences of *Lilium martagon* for the phytocoenoses it occupies. Two of the sites we studied were located in the oak-hornbeam forest *Tilio cordatae–Carpinetum betuli*, while the third site was in the mountain beech forest *Dentario glandulosae–Fagetum*.

Natural forest phytocoenoses with *Lilium martagon* are characterised by high species richness, and a particularly high share of characteristic species from the order *Fagetalia*. In such communities, *Lilium martagon* populations are usually more numerous and stable [23,30,32,85,87]. We observed similar relationships in our studies. The highest species richness and the highest diversity indices were recorded in Hrabeński Forest, where well-developed and properly preserved natural habitats are protected [21]. The smaller number of species and lower diversity indices on Mount Chełm can be explained by the habitat type. The Carpathian beech forest *Dentario glandulosae–Fagetum* occurs here, which, unlike oak-hornbeam forests, is characterised by lower species richness [20]. The lowest Shannon–Wiener diversity index in the case of Wolski Forest can be explained by the high intensity of anthropopressure at this site, resulting in heavy trampling of the forest floor and the penetration of synanthropic species [89]. Other authors have also pointed out the negative impact of anthropopressure manifesting itself with a decrease in abundance or complete disappearance of the population of *Lilium martagon* [58,62].

### 4.2. The Spatial and Temporal Variability of Abundance and Structure of Population Lilium martagon

The greatest abundance of population of *Lilium martagon* was found at site 3 (Hrabeński Forest), where the highest values of soil moisture, light intensity at ground level, and height of plant cover were noticed. Despite the substantial share of generative stems in population 3 during whole study period, exceeding those recorded in populations 1 and 2, as well as many others [23,24,60,61,62,63], a lack of juvenile stems was noted. Considering the successful germination of the seed of *Lilium martagon* in laboratory conditions [90], the absence of juvenile stems in population 3 presumably might be caused by the dense vegetation cover and the absence of gaps in continuous plant cover acting as safe sites for seedling recruitment *sensu* Harper et al. [91]. The presence of juvenile stems in population 1 and population 2, exceeding values recorded by other authors [29,65], might be linked to the occurrence of openings in the plant cover, causing, among other things, the death of senile plants or ramets (in the case of clonal species), as well as disturbances caused by the natural activity of forest mammals, such as burrowing and digging. Furthermore, the presence of natural gaps at site 2 might be linked to the occurrence of shallow soil with a significant content of skeletal elements, such as gravel, cobbles, stones, and rock fragments, whose spontaneous subsidence causes the removal of plants. Additionally, the high tourist pressure documented at site 1 (Wolski Forest) by Kostrakiewicz-Gierałt et al. [89] might contribute to gap appearance. The aforementioned authors argued that trampling by people frequently accompanied by dogs might affect the creation of gaps in the plant cover of the forest floor.

The recorded temporal differences in the abundance of populations and their structure might be connected, on the one hand, with the recruitment of new individuals in a generative or vegetative way and their subsequent growth, and, on the other hand, with the prolonged bulb dormancy or mortality of individuals. The latter might be a consequence of the natural senescence process of individuals or be triggered by external factors such as people or herbivorous animal activity. The recreational activities of people, such as walking, jogging or biking, as well as divergence from formal trails and spontaneous creation of informal pathways, recorded among others in Wolski Forest [89], might contribute to damage or/and mortality of juvenile stems of *Lilium martagon*. Additionally, the blooming stems of *Lilium martagon* might be picked, while the bulbs might be dug out by visitors due to the ornamental value. Furthermore, herbivorous animals including wild boars, whose occurrence has been documented in Wolski Forest [92], might contribute to bulbs being dug up, affecting the mortality of individuals of the studied species. Moreover, deer, whose presence has been confirmed on Mount Chełm and in Hrabeński Forest (personal observation) may browse on the stems of *Lilium martagon*, contributing to a decline in the population abundance. The damage and mortality of individuals of *Lilium martagon* might also be caused by the activity of the scarlet lily beetle (*Lilioceris lilii*), traces of whose feeding activity were recorded in the studied populations, as well as in many other locations [23,65].

Furthermore, the recorded temporal differences in the abundance of populations and their structure might be linked to weather conditions. The greatest share of juvenile stems in population 1 observed in the year 2019 comparing to other study seasons might be connected with favourable weather conditions, such as considerable precipitation during the meteorological spring (especially in May) and substantial values of air temperatures during the meteorological spring and summer (particularly in June). Such observations remain in accordance with the findings of Truchan and Sobisz [64], who claimed that substantial precipitation and air temperature from May to July favour the growth and development of *Lilium martagon* individuals. On the other hand, in the year 2019 in populations 2 and 3, the lowest number of stems in the whole study period were recorded despite substantial precipitation and considerable air temperatures. Additionally, the share of juvenile stems in population 2 was rather similar to the share noted in other study years.

### 4.3. Spatial Variability of Traits of Stems of Lilium martagon

The highest number of immature and virginile stems with the greatest number of whorl leaves, as well as the substantial height of generative stems and number of whorl leaves observed in population 3 might be the result of growing in conditions of lateral shading by adjacent plants. As stated by Fiorucci and Fankhauser [93], shading by neighbouring herbaceous species reduces energy for photosynthesis and contributes to stem elongation. Under crowding conditions, the height increment is advantageous because it allows neighbours to be overtopped and contributes to more effective light interception. At the same time, it should be noted that gaps in tree canopy enabled successful light assimilation by overgrowing adjacent plants of *Lilium martagon* stems and might explain the lowest dimensions of whorl and spiral leaves in population 3. The lowest height of immature and virginile stems recorded in population 1 and generative stems noticed in population 2 might be caused by their over shading by the canopy of surrounding trees, which might contribute to elongation suppression. Such findings seem to correspond with the observations of Miciniak and Zątek [24], who demonstrated that individuals of *Lilium martagon* growing in the dry-mesic oak forest reached greater height on average than those occurring in the oak-hornbeam forest. Also, the findings of Pindel [63] demonstrated that the stems of *Lilium martagon* are much shorter in localities where light availability is slight than in not shaded stands.

Although the increase in flower number observed from population 2 via population 3 to population 1 increased the visibility of inflorescences and chances for successful pollination and seed production, it should be pointed out that the recorded mean number of flowers in the studied populations was rather low and corresponds with the observations of Bednorz [23] and Gazda and Gazda [29], while the greater number of flowers observed in the year 2019 in population 1 was close to mean values which were noted by Truchan [65] in a manor park. At the same time, it is worth adding that in populations occurring in manor parks [64] and in grasslands [10,69], the mean values of number of flowers per stems are much greater.

### 4.4. The Temporal Variability of Traits of Stems of Lilium martagon

The significantly greater values of selected individual traits observed in the year 2019 in immature stems (height of stems in populations 2 and 3, number, length and width of whorl leaves in population 1) and in generative stems (height of stems, number of flowers and whorls in population 1, number and length of whorl leaves in population 3) might suggest the favourable impact of weather conditions (specifically substantial precipitation and air temperatures) during the meteorological spring and summer on the growth of *Lilium martagon* stems. The aforementioned findings seem to correspond with the experimental studies of Khashroum et al. [94], who demonstrated the positive effect of substantial water irrigation among others on the length of *Lilium martagon* stems and flower number.

On the other hand, performed investigations showed that several traits did not exhibit temporal variability at least in one population. The lack of a unified trend in the studied populations might suggest the occurrence of site-specific temporal variability of individual traits. Micro-environmental soil conditions, such as nutrient availability (determined by the interplay of various nutrients and soil characteristics such as pH, texture and organic matter) might fluctuate during the growing season and may differ among sites. The seasonal fluctuations in nutrient availability were evidenced by Díaz-Raviña et al. [95], while variability among study sites was confirmed among others in pine forests [96] and beech forests [97].

## 5. Conclusions

The observed spatial variability of the studied populations allows the conclusion to be drawn that substantial soil moisture, considerable light intensity at ground level, as well as slightly acidic soil pH and moderate nitrogen content favour the development and growth of *Lilium martagon* individuals. On the other hand, factors negatively affecting its growth are represented by the lack of microsites suitable for seedling recruitment, over shading of stems by the tree canopy, the presence of shallow soils, as well as human and herbivorous animal activity contributing to damage of the above- and/or belowground parts of individuals. The study observations suggested that considerable precipitation during the meteorological spring (especially in May) and substantial values of air temperatures during the meteorological spring and summer (particularly in June) contribute to the growth of *Lilium martagon* stems. However, the lack of a unified trend in all the studied populations might indicate the occurrence of site-specific temporal variability of individual traits.

Moreover, the obtained results allow us to evaluate the state of the observed populations. The most abundant population, population 3, located in an oak-hornbeam forest *Tilio cordatae–Carpinetum betuli* in Hrabeński Forest, presents the greatest prospects for persistence in the occupied site. However, it is highly necessary for artificial openings to be made in the dense plant cover to create suitable microsites for seedling recruitment and development of juvenile individuals. Although stems representing all developmental stages were noticed in population 1, situated in the oak-hornbeam forest *Tilio cordatae–Carpinetum betuli* in Wolski Forest, and in population 2 in the beech forest *Dentario glandulosae–Fagetum* in Mount Chełm, the state of these populations seems unsatisfactory due to their much lower abundance. Furthermore, in more shaded areas, it is recommended to remove individual trees and shrubs to ensure 5–25% light availability for the herb layer.

All studied populations are located in protected areas, although their protection regimes vary. In the case of Mount Chełm Nature Reserve and Hrabeński Forest (Natura 2000 Hrabeński Forest PLH180039 site), passive conservation protection appears to be an effective strategy for the preservation of the *Lilium martagon* population, especially since these sites are located far from built-up areas. However, in Wolski Forest (Bielańsko-Tyniecki Landscape Park), a low protection regime is ineffective in counteracting the increased human impact. Unfortunately, the proximity of a large urban area contributes to the excessive trampling and destruction of vegetation, including the *Lilium martagon* population. Therefore, it is necessary to implement constant monitoring and channel tourist traffic through appropriately designated paths and trails that would bypass the *Lilium martagon* site.

Considering the obtained results, it can be concluded that long-term (covering at least several years) observations of populations of *Lilium martagon* should be continued. Such laborious, demanding, and time-consuming investigations provide the essential data supplementing the current state of knowledge and are crucial for better protection of this rare species. Of particular value would be comparisons of population and individual traits of *Lilium martagon* occurring in different habitats, including forests, grasslands, and anthropogenic localities.

## Figures and Tables

**Table 1:** Characteristics of vegetation at study site 1 (Wolski Forest), study site 2 (Mount Chełm) and study site 3 (Hrabeński Forest).

Sites	Site 1 Wolski Forest	Site 2 Mount Chełm	Site 3 Hrabeński Forest
Community	*Tilio cordatae–Carpinetum betuli*	*Dentario glandulosae–Fagetum*	*Tilio cordatae–Carpinetum betuli*
Average value of tree layer cover [%]	84	77	88
Average value of shrub layer cover [%]	45	5	34
Average value of herb layer cover [%]	68	76	91
Dominant species in the tree layer	*Tilia cordata* *Quercus robur*	*Fagus sylvatica* *Acer pseudoplatanus*	*Carpinus betulus* *Acer pseudoplatanus* *Quercus robur*
Dominant species in the shrub layer	*Acer pseudoplatanus* *Acer platanoides* *Corylus avellana*	*-*	*Tilia cordata* *Fagus sylvatica* *Sambucus nigra*
Dominant species in the herb layer	*Stellaria holostea* *Asarum europaeum* *Galeobdolon luteum* *Polygonatum multiflorum* *Anemone nemorosa*	*Dentaria glandulosa* *Galeobdolon luteum* *Rubus hirtus*	*Stellaria holostea* *Asarum eropaeum* *Galeobdolon luteum* *Galium odoratum* *Lilium martagon* *Primula elatior* *Pulmonaria obscura* *Aegopodium podagraria*

**Table 2:** Mean (±SD) height, number of leaves in the whorl, length and width of the longest leaf in the whorl of immature stems in population 1 located in Wolski Forest, population 2 located on Mount Chełm, and population 3 located in Hrabeński Forest in the years 2018–2023. The asterisks indicate statistical significance level: * *p* ≤ 0.05, ** *p* < 0.01, *** *p* < 0.001, ns-not significant. The different letters below H values indicate significant differences among populations.

Year	Population	Height of Stem [cm]	Number of Leaves in the Whorl	Length of the Longest Leaf in the Whorl [mm]	Width of the Longest Leaf in the Whorl [mm]
2018	1	12.50 (±3.75)	6.10 (±1.20) ^a^	97.90 (±32.49)	35.70 (±6.20)
	2	12.65 (±4.56)	7.85 (±1.81) ^a^	102.56 (±19.08)	36.37 (±10.10)
	3	15.86 (±2.10)	8.09 (±1.76) ^b^	112.55 (±17.80)	37.55 (±6.17)
	Kruskal–Wallis H test	5.95 ^ns^	8.65 *	2.13 ^ns^	0.51 ^ns^
2019	1	17.56 (±4.44) ^b^	9.40 (±2.52) ^b^	139.28 (±15.52) ^c^	39.72 (±6.30)
	2	11.53 (±3.38) ^a^	6.73 (±1.95) ^a^	97.00 (±18.88) ^a^	35.67 (±5.23)
	3	18.77 (±4.64) ^b^	8.95 (±1.86) ^b^	119.14 (±12.39) ^b^	37.36 (±5.19)
	Kruskal–Wallis H test	31.63 ***	17.54 ***	42.97 ***	5.66 ^ns^
2020	1	11.40 (±3.96)	5.58 (±1.61) ^a^	101.46 (±25.90)	33.00 (±8.32) ^a^
	2	11.88 (±3.54)	6.92 (±1.70) ^b^	101.69 (±19.96)	32.96 (±6.72) ^a^
	3	13.71 (±4.15)	7.79 (±1.47) ^b^	95.17 (±12.92)	38.75 (±6.25) ^b^
	Kruskal–Wallis H test	4.29 ^ns^	19.13 ***	3.13 ^ns^	10.13 **
2021	1	12.33 (±7.64) ^a^	5.23 (±2.35) ^a^	102.77 (±24.18) ^a^	38.61 (±16.56) ^a^
	2	17.12 (±4.21) ^b^	7.43 (±1.96) ^b^	119.05 (±17.65) ^b^	45.43 (±8.08) ^b^
	3	14.73 (±4.53) ^ab^	8.69 (±2.43) ^b^	94.15 (±15.02) ^a^	34.46 (±7.60) ^a^
	Kruskal–Wallis H test	13.36 **	24.20 ***	20.59 ***	18.18 ***
2022	1	12.30 (±2.86) ^a^	6.20 (±1.42)	99.57 (±29.99)	32.33 (±5.30) ^a^
	2	16.37 (±5.22) ^b^	6.63 (±2.67)	108.26 (±20.31)	40.74 (±6.56) ^b^
	3	16.33 (±4.86) ^b^	7.30 (±2.08)	103.70 (±18.23)	32.96 (±6.56) ^a^
	Kruskal–Wallis H test	9.24 **	2.69 ^ns^	1.05 ^ns^	14.69 ***
2023	1	11.02 (±3.27) ^a^	5.78 (±1.80) ^a^	111.44 (±22.41) ^ab^	34.48 (±7.16) ^a^
	2	16.30 (±4.77) ^b^	6.74 (±1.91) ^a^	119.57 (±12.66) ^b^	44.09 (±7.85) ^b^
	3	18.15 (±4.62) ^b^	7.77 (±1.74) ^b^	96.31 (±12.45) ^a^	38.77 (±8.33) ^ab^
	Kruskal–Wallis H test	24.55 ***	9.90 **	12.91 **	15.27 ***

**Table 3:** Mean (±SD) height, number of leaves in the lower whorl, length and width of the longest leaf in the whorl of virginile stems in the observed populations in 2018–2023. The lack of data for population 3 in 2018 and in population 2 in 2019 is caused by the absence of virginile stems within the study patches. The population localities and the description of statistical significance levels—as in Table 2.

Year	Population	Height of Stem [cm]	Number of Leaves in the Lower Whorl	Length of Leaves in the Lower Whorl [mm]	Width of Leaves in the Lower Whorl [mm]
2018	1	13.14 (±4.41)	6.57 (±1.72)	102.00 (±12.88)	36.43 (±9.24)
	2	24.83 (±4.19)	7.33 (±1.15)	115.67 (±9.29)	43.67 (±2.08)
	3	-	-	-	-
	Mann–Whitney U test	0.00 *	7.50 ^ns^	4.0 ^ns^	6.50 ^ns^
2019	1	19.40 (±4.49)	7.80 (±1.79)	126.60 (±13.85)	29.40 (±0.89)
	2	-	-	-	-
	3	26.38 (±5.48)	9.75 (±3.20)	118.88 (±15.66)	37.38 (±7.95)
	Mann–Whitney U test	6.0 *	13.00 ^ns^	14.00 ^ns^	U = 5.0 *
2020	1	15.17 (±3.71) ^a^	6.17 (±1.17) ^a^	110.83 (±13.20)	35.00 (±6.60)
	2	20.13 (±2.25) ^ab^	6.25 (±0.50) ^ab^	109.50 (±8.35)	45.75 (±6.65)
	3	22.17 (3.72) ^b^	8.33 (1.86) ^b^	109.33 (15.53)	34.67 (5.35)
	Kruskal–Wallis H test	7.20 *	7.51 *	0.5 ^ns^	5.96 ^ns^
2021	1	22.86 (±3.39)	6.57 (±1.90)^a^	128.71 (±11.83)	38.43 (±7.46)
	2	23.06 (±3.97)	6.44 (±1.01)^a^	125.22 (±10.24)	42.11 (±4.23)
	3	31.25 (±6.40)	11.00 (±1.83)^b^	109.75 (±8.14)	41.00 (±6.22)
	Kruskal–Wallis H test	5.84 ^ns^	8.67 *	5.97 ^ns^	1.07 ^ns^
2022	1	19.58 (±6.40)	5.54 (±0.78)^a^	117.92 (±14.92)	34.62 (±6.28) ^a^
	2	26.00 (±5.08)	6.91 (±1.30)^ab^	115.64 (±15.69)	43.73 (±7.81) ^b^
	3	27.14 (±5.61)	8.29 (±2.43)^b^	103.43 (±17.89)	34.14 (±5.58) ^a^
	Kruskal–Wallis H test	7.21 ^ns^	9.38 **	3.42 ^ns^	8.24 *
2023	1	19.67 (±2.08)	5.33 (±0.58)	123.00 (±14.73)	38.00 (±2.65)
	2	23.14 (±3.02)	7.57 (±1.40)	118.57 (±6.92)	45.57 (±6.13)
	3	27.00 (±5.66)	8.50 (±2.12)	117.50 (±7.78)	37.00 (±1.41)
	Kruskal–Wallis H test	4.23 ^ns^	5.80 ^ns^	0.9 ^ns^	5.67 ^ns^

**Table 4:** Mean (±SD) height, number of flowers, number of whorls of generative stems in the observed populations in 2018–2023. The population localities and the description of statistical significance levels—as in Table 2.

Year	Population	Height of Stem [cm]	Number of Flowers	Number of Whorls
2018	1	78.39 (±20.71) ^b^	3.56 (±1.82) ^b^	2.28 (±0.89) ^b^
	2	68.75 (±13.06) ^a^	2.60 (±1.40) ^a^	1.40 (±0.50) ^a^
	3	76.29 (±11.05)^b^	3.26 (±1.41) ^b^	1.42 (±0.51) ^a^
	Kruskal–Wallis H test	12.43 **	13.04 **	14.18 ***
2019	1	97.00 (±11.34) ^b^	6.20 (±1.10) ^b^	3.00 (±0.71) ^b^
	2	55.27 (±14.42) ^a^	2.27 (±0.90) ^a^	1.73 (±0.47) ^a^
	3	77.45 (±11.76) ^b^	3.10 (±1.09) ^a^	1.83 (±0.38) ^a^
	Kruskal–Wallis H test	20.40 ***	16.34 ***	15.96 ***
2020	1	67.10 (±24.40) ^ab^	2.60 (±2.03) ^a^	1.37 (±0.49) ^a^
	2	59.79 (±12.27) ^a^	2.17 (±0.93) ^a^	1.62 (±0.49) ^ab^
	3	75.20 (±12.30) ^b^	3.80 (±1.65) ^b^	1.83 (±0.46) ^b^
	Kruskal–Wallis H test	15.57 **	20.65 ***	13.81 **
2021	1	61.17 (±13.60) ^a^	2.89 (±1.32)	1.44 (±0.51)
	2	61.53 (±9.98) ^a^	2.43 (±0.82)	1.77 (±0.50)
	3	70.27 (±11.18) ^b^	3.03 (±1.16)	1.53 (±0.51)
	Kruskal–Wallis H test	12.47 **	5.90 ^ns^	4.97 ^ns^
2022	1	81.33 (±13.65)	1.67 (±0.58)^a^	1.67 (±0.58)
	2	72.93 (±11.32)	2.68 (±0.90)^a^	1.82 (±0.39)
	3	81.30 (±14.61)	4.13 (±1.38)^b^	2.10 (±0.66)
	Kruskal–Wallis H test	4.68 ^ns^	19.67 ***	3.98 ^ns^
2023	1	58.67 (±9.87) ^ab^	2.33 (±0.58) ^a^	1.67 (±0.58)
	2	56.30 (±11.29) ^a^	2.40 (±0.84) ^a^	1.70 (±0.48)
	3	85.43 (±17.08) ^b^	4.53 (±2.21) ^b^	2.07 (±0.58)
	Kruskal–Wallis H test	18.07 ***	11.60 **	3.80 ^ns^

**Table 5:** Mean (±SD) number of leaves in the lowest whorl, length and width of the longest leaf in the lowest whorl, number of spiral leaves, length and width of the spiral leaf above the highest whorl of generative stems in the observed populations in 2018–2023. The population localities and the description of statistical significance levels—as in Table 2.

Year	Population	Number of Leaves in the Lowest Whorl	Length of the Longest Leaf in the Lowest Whorl [mm]	Width of the Longest Leaf in the Lowest Whorl [mm]	Number of Spiral Leaves	Length of Spiral Leaf Above the Highest Whorl [mm]	Width of the Spiral Leaf Above the Highest Whorl [mm]
2018	1	6.50 (±1.72) ^a^	129.72 (±19.04)	35.72 (±6.80)	7.17 (±2.15) ^b^	103.33 (±12.01)	26.17 (±6.95)
	2	7.50 (±1.66) ^a^	116.93 (±12.53)	39.53 (±5.81)	5.20 (±2.02) ^a^	94.73 (±16.35)	25.13 (±7.89)
	3	9.00 (±1.76) ^b^	119.42 (±18.68)	39.63 (±6.23)	5.26 (±1.48) ^a^	94.63 (±14.42)	23.68 (±7.54)
	Kruskal–Wallis H test	27.02 ***	4.56 ^ns^	3.25 ^ns^	12.71 *	4.28 ^ns^	4.39 ^ns^
2019	1	6.60 (±2.70) ^a^	147.80 (±22.86)	40.60 (±9.86)	7.20 (±2.77) ^b^	102.60 (±9.13)	20.20 (±1.79)
	2	7.45 (±2.38) ^a^	123.27 (±16.03)	39.36 (±5.52)	3.73 (±1.10) ^a^	89.09 (±18.24)	23.45 (±8.86)
	3	9.47 (±2.69) ^b^	126.13 (±11.99)	38.63 (±6.89)	4.73 (±1.91) ^ab^	83.37 (±19.35)	16.73 (±5.94)
	Kruskal–Wallis H test	10.95 **	5.33 ^ns^	0.40 ^ns^	10.30 **	4.99 ^ns^	5.81 ^ns^
2020	1	5.57 (±1.43)	119.57 (±13.68)	32.93 (±6.86)	8.50 (±2.05) ^b^	112.43 (±17.07) ^b^	30.53 (±7.62) ^b^
	2	6.86 (±1.64)	110.69 (±16.91)	34.93 (±8.90)	3.59 (±1.45) ^a^	83.59 (±22.41) ^a^	21.38 (±9.20) ^a^
	3	8.00 (±1.51)	118.50 (±10.11)	35.43 (±9.17)	4.30 (±1.32) ^a^	89.10 (±15,34) ^a^	21.83 (±17.25) ^a^
	Kruskal–Wallis H test	4.73 ^ns^	4.17 ^ns^	2.94 ^ns^	55.90 ***	28.98 ***	23.87 ***
2021	1	6.00 (±1.37) ^a^	124.50 (±19.31) ^b^	36.78 (±6.23) ^a^	6.83 (±3.47) ^b^	105.72 (±25.77)^b^	25.89 (±11.25) ^b^
	2	7.27 (±1.57) ^b^	125.63 (±13.84) ^b^	42.70 (±5.62) ^b^	3.80 (±1.30) ^a^	95.37 (±13.02)^b^	23.07 (±5.62) ^b^
	3	8.37 (±1.67) ^b^	109.67 (±14.92) ^a^	36.73 (±7.23) ^a^	4.43 (±1.91) ^a^	81.83 (±20.05)^a^	18.00 (±9.01) ^a^
	Kruskal–Wallis H test	19.41 ***	15.96 ***	13.97 ***	15.51 ***	13.06 **	9.11 *
2022	1	6.33 (±0.58) ^a^	134.00 (±19.31) ^b^	40.00 (±8.66) ^ab^	7.67 (±0.58) ^b^	113.33 (±7.64)^b^	28.33 (±2.89) ^b^
	2	7.21 (±1.91) ^a^	131.39 (±18.40) ^b^	43.00 (±6.73) ^b^	4.11 (±1.50) ^a^	95.50 (±16.51)^b^	23.04 (±6.60) ^ab^
	3	9.37 (±3.80) ^b^	119.67 (±16.10) ^a^	36.33 (±6.29) ^a^	4.77 (±2.31) ^a^	84.90 (±14.16)^a^	18.90 (±4.71) ^a^
	Kruskal–Wallis H test	10.71 **	8.69 *	12.28 **	8.37 *	11.24 **	9.44 **
2023	1	7.00 (±0.00)	120.67 (±29.96)	33.00 (±14.80) ^ab^	4.00 (±1.00)	97.67 (±28.99)	23.00 (±9.54)
	2	8.40 (±1.71)	125.20 (±12.00)	41.30 (±5.01) ^b^	4.70 (±1.42)	88.60 (±22.07)	21.10 (±7.64)
	3	8.33 (±1.65)	118.80 (±15.56)	35.03 (±5.51) ^a^	4.73 (±1.60)	83.13 (±12.91)	18.03 (±5.22)
	Kruskal–Wallis H test	3.33 ^ns^	2.11 ^ns^	6.87 *	0.57 ^ns^	3.36 ^ns^	2.85 ^ns^

## Data Availability

The original contributions presented in this study are included in the article. Further inquiries can be directed to the corresponding author.

## Supplementary Materials

The following supporting information can be downloaded at: https://www.mdpi.com/article/10.3390/biology15050398/s1, Supplementary Figure S1: Mean height (A), number of leaves in the whorl (B), length (C), and width (D) of the longest leaf in the whorl of immature stems in population 1 located in Wolski Forest (P1), population 2 located in Mount Chełm (P2), and population 3 located in Hrabeński Forest (P3) in the years 2018–2023. The asterisks mean statistical significance level: * *p* ≤ 0.05, ** *p* < 0.01, *** *p* < 0.001 (The Kruskal–Wallis H test). The different letters below H values mean the significant differences among populations; Supplementary Figure S2: Mean height (A), number of leaves in the lower whorl (B), length (C), and width (D) of the longest leaf in the lower whorl of virginile stems in the observed populations. The explanations of abbreviations P1–P3 and description of statistical significance levels—as in Figure S1. Supplementary Figure S3: Mean height (A), number of flowers (B), and number of whorls (C) in generative stems in the observed populations. The explanations of abbreviations P1–P3 and description of statistical significance levels—as in Figure S1. Supplementary Figure S4: Mean number of leaves in the lowest whorl (A), length (B), and width (C) of the longest leaf in the lowest whorl in generative stems in the observed populations. The explanations of abbreviations P1–P3 and description of statistical significance levels—as in Figure S1. Supplementary Figure S4: Mean number of leaves in the lowest whorl (A), length (B), and width (C) of the longest leaf in the lowest whorl in generative stems in the observed populations. The explanations of abbreviations P1-P3 and description of statistical significance levels—as in Figure S1. Supplementary Figure S5: Mean number of spiral leaves (A), length (B), and width (B) of spiral leaf above the highest whorl in generative stems in the observed populations. The explanations of abbreviations P1–P3 and description of statistical significance levels—as in Figure S1. Supplementary Table S1: Values of the d-Cohen coefficient regarding the height, number of leaves in the whorl, and length and width of the longest leaf in the whorl of immature stems among pairs of study years in population 1 located in Wolski Forest, population 2 located on Mount Chełm, and population 3 located in Hrabeński Forest. Moderate (absolute value d > 0.5) and substantial (absolute value d > 0.8) differences in the d-Cohen coefficient among the populations are highlighted in bold. Supplementary Table S2: Values of the d-Cohen coefficient regarding the height, number of leaves in the whorl, and length and width of the longest leaf in the whorl of virginile stems among pairs of study years in the observed populations. Population localities and description of d-Cohen coefficient values—as in Table S1. Supplementary Table S3: Values of the d-Cohen coefficient regarding the height, number of flowers, and number of whorls of generative stems among pairs of study years in the observed populations. Population localities and description of d-Cohen coefficient values—as in Table S1. Supplementary Table S4: Values of the d-Cohen coefficient regarding the number of leaves in the lowest whorl, length and width of the longest leaf in the lowest whorl, number of spiral leaves, and length and width of the spiral leaf above the highest whorl of generative stems among pairs of study years in the observed populations. Population localities and description of d-Cohen coefficient values—as in Table S1.

## Appendix A

**Table A1:** Values of d- the Cohen coefficient regarding the values of the Shannon index, Simpson index, Pielou index and the number of species, soil moisture (%), soil pH, height of plant cover, and light intensity at ground level (lx) at study site 1 (Wolski Forest), study site 2 (Mount Chełm), and study site 3 (Hrabeński Forest). The moderate (absolute value d > 0.5) and substantial (absolute value d > 0.8) differences in d-Cohen coefficient calculated for pairs of study sites are highlighted in bold.

Compared Study Sites	Shannon Index	Simpson Index	Pielou Index	Number of Species	Soil Moisture	Soil Reaction	Height of Plant Cover	Light Intensity
1–2	−0.05	0.00	−0.13	0.09	−0.40	**0.77**	−0.28	−0.39
1–3	**−1.09**	**−0.84**	**−0.61**	**−1.45**	**−2.71**	0.14	−0.46	**−1.53**
2–3	**−1.13**	**−0.69**	−0.30	**−1.89**	**−1.91**	**−0.97**	−0.30	**−1.78**

**Table A2:** Values of the d-Cohen coefficient regarding the values of Ellenberg indicators of light, temperature, continentalism, moisture, soil reaction, and nitrogen content at the study sites. Localities of the study sites and description of d-Cohen coefficient values—as in Table A1.

Compared Study Sites	Light	Temperature	Continentalism	Moisture	Soil Reaction	Nitrogen Content
1–2	−0.39	**1.60**	**1.41**	**0.66**	**−1.38**	0.39
1–3	**−1.53**	0.32	−0.18	**0.67**	**−0.76**	0.35
2–3	**−1.78**	**−1.33**	**−1.92**	−0.13	0.22	−0.09

**Table A3:** Values of the d-Cohen coefficient regarding the height, number of leaves in the whorl, and length and width of the longest leaf in the whorl of immature stems in population 1 located in Wolski Forest, population 2 located on Mount Chełm, and population 3 located in Hrabeński Forest. The moderate (absolute value d > 0.5) and substantial (absolute value d > 0.8) differences calculated for pairs of populations are highlighted in bold.

Year	Compared Populations	Height of Stem [cm]	Number of Leaves in the Whorl	Length of the Longest Leaf in the Whorl [mm]	Width of the Longest Leaf in the Whorl [mm]
2018	1–2	−0.1	**−0.56**	−0.09	−0.04
	1–3	**−0.57**	**−0.67**	−0.29	−0.14
	2–3	**−0.58**	−0.06	−0.27	−0.06
2019	1–2	**0.70**	**0.59**	**1.22**	0.35
	1–3	−0.13	0.10	**0.72**	0.20
	2–3	**−0.90**	**−0.58**	**−0.66**	−0.16
2020	1–2	−0.06	−0.37	−0.005	0.002
	1–3	−0.28	**−0.73**	0.16	−0.39
	2–3	−0.23	−0.27	0.19	−0.44
2021	1–2	−0.40	−0.50	−0.38	−0.27
	1–3	−0.19	**−0.72**	0.21	0.17
	2–3	0.27	−0.28	**0.76**	**0.69**
2022	1–2	−0.50	−0.10	−0.17	**−0.70**
	1–3	**−0.52**	−0.31	−0.08	−0.05
	2–3	0.003	−0.14	0.11	**0.59**
2023	1–2	**−0.65**	−0.25	−0.23	**−0.64**
	1–3	**−0.90**	**−0.56**	**0.53**	0.27
	2–3	−0.19	−0.28	**0.92**	0.32

**Table A4:** Values of the d-Cohen coefficient regarding the number of leaves in the lower whorl and length and width of the longest leaf in the whorl of virginile stems in the observed populations. Population localities and description of d-Cohen coefficient values—as in Table A3.

Year	Compared Populations	Height of Stem [cm]	Number of Leaves in the Lower Whorl	Length of Leaves in the Lower Whorl [mm]	Width of Leaves in the Lower Whorl [mm]
2018	1–2	−0.36	−0.27	**−0.62**	**−0.64**
2019	1–3	−1.34	**−0.65**	**−0.59**	−0.06
2020	1–2	**−0.83**	−0.05	0.06	**−0.81**
	1–3	**−0.94**	**−0.71**	0.05	0.03
	2–3	**−7.08**	**−6.17**	**−9.17**	**−6.70**
2021	1–2	−0.03	0.04	0.16	−0.32
	1–3	**−0.86**	**−1.19**	**0.95**	−0.19
	2–3	**−0.79**	**−1.60**	**0.84**	0.11
2022	1–2	**−0.56**	**−0.66**	0.07	**−0.65**
	1–3	**−0.63**	**−0.86**	0.44	0.04
	2–3	−0.11	−0.37	0.36	**0.72**
2023	1–2	**−0.68**	**−1.13**	0.20	**−0.86**
	1–3	**−0.95**	**−1.17**	0.24	0.25
	2–3	0.44	0.26	−0.07	**−1.14**

**Table A5:** Values of the d-Cohen coefficient regarding the height, number of flowers, and number of whorls of generative stems in the observed populations. Population localities and description of d-Cohen coefficient values—as in Table A3.

Year	Compared Populations	Height of Stem [cm]	Number of Flowers	Number of Whorls
2018	1–2	0.49	0.30	**0.63**
	1–3	0.07	0.09	**0.61**
	2–3	−0.31	−0.24	−0.02
2019	1–2	**0.66**	0.47	0.40
	1–3	0.03	0.16	0.35
	2–3	−0.33	−0.25	−0.05
2020	1–2	0.20	0.14	−0.26
	1–3	−0.22	−0.33	−0.49
	2–3	**−5.49**	**−2.32**	**−3.62**
2021	1–2	−0.02	0.21	−0.32
	1–3	−0.37	−0.06	−0.09
	2–3	−0.41	−0.30	0.23
2022	1–2	**0.54**	**−0.68**	−0.16
	1–3	0.00	**−1.26**	−0.35
	2–3	−0.32	**−0.64**	−0.26
2023	1–2	0.11	−0.05	−0.03
	1–3	**−0.99**	**−0.79**	−0.34
	2–3	**1.03**	**0.70**	0.34

**Table A6:** Values of the d-Cohen coefficient regarding the number of leaves in the lowest whorl, length and width of the longest leaf in the lowest whorl, number of spiral leaves, and length and width of the spiral leaf above the highest whorl of generative stems in the observed populations. Population localities and description of d-Cohen coefficient values—as in Table A3.

Year	Compared Populations	Number of Leaves in the Lowest Whorl	Length of the Longest Leaf in the Lowest Whorl [mm]	Width of the Longest Leaf in the Lowest Whorl [mm]	Number of Spiral Leaves	Length of Spiral Leaf Above the Highest Whorl [mm]	Width of the Spiral Leaf Above the Highest Whorl [mm]
2018	1–2	−0.30	0.41	−0.30	0.47	0.30	0.07
	1–3	**−0.72**	0.27	−0.30	**0.52**	0.33	0.17
	2–3	−0.44	−0.08	−0.01	−0.02	0.00	0.09
2019	1–2	−0.23	0.18	−0.30	1.06	0.47	0.17
	1–3	**−0.67**	0.12	−0.21	**0.60**	**0.64**	**0.73**
	2–3	−0.20	−0.02	0.02	−0.18	0.05	0.23
2020	1–2	−0.42	0.29	−0.13	**1.40**	**0.73**	**0.54**
	1–3	**−0.83**	0.04	−0.16	**1.25**	**0.72**	0.35
	2–3	**−4.72**	**−8.48**	**−3.89**	**−2.85**	**−4.57**	**−1.63**
2021	1–2	−0.43	−0.03	−0.50	**0.64**	0.27	0.17
	1–3	**−0.78**	0.43	0.00	0.45	**0.52**	0.39
	2–3	−0.34	**0.56**	0.46	−0.20	0.41	0.35
2022	1–2	−0.35	0.07	−0.19	**1.71**	**0.74**	**0.56**
	1–3	**−0.69**	0.40	0.25	**1.00**	**1.30**	**1.24**
	2–3	−0.38	0.34	**0.51**	−0.17	0.35	0.37
2023	1–2	**−0.82**	−0.11	−0.42	−0.29	0.18	0.11
	1–3	**−0.81**	0.04	−0.10	−0.28	0.35	0.34
	2–3	−0.02	−0.23	**−0.60**	0.01	−0.16	−0.24
