# Supplementary material for: Spatio-Temporal Variability Description of the Rare Species Lilium martagon L. in Different Habitat Conditions
Source: Biology (Basel). 2026 Feb 28;15(5):398. doi: 10.3390/biology15050398 (PMC12985253; doi:10.3390/biology15050398)
Supplement: Supplementary file 1 [file biology-15-00398-s001.zip › biology-4133583-supplementary.pdf]

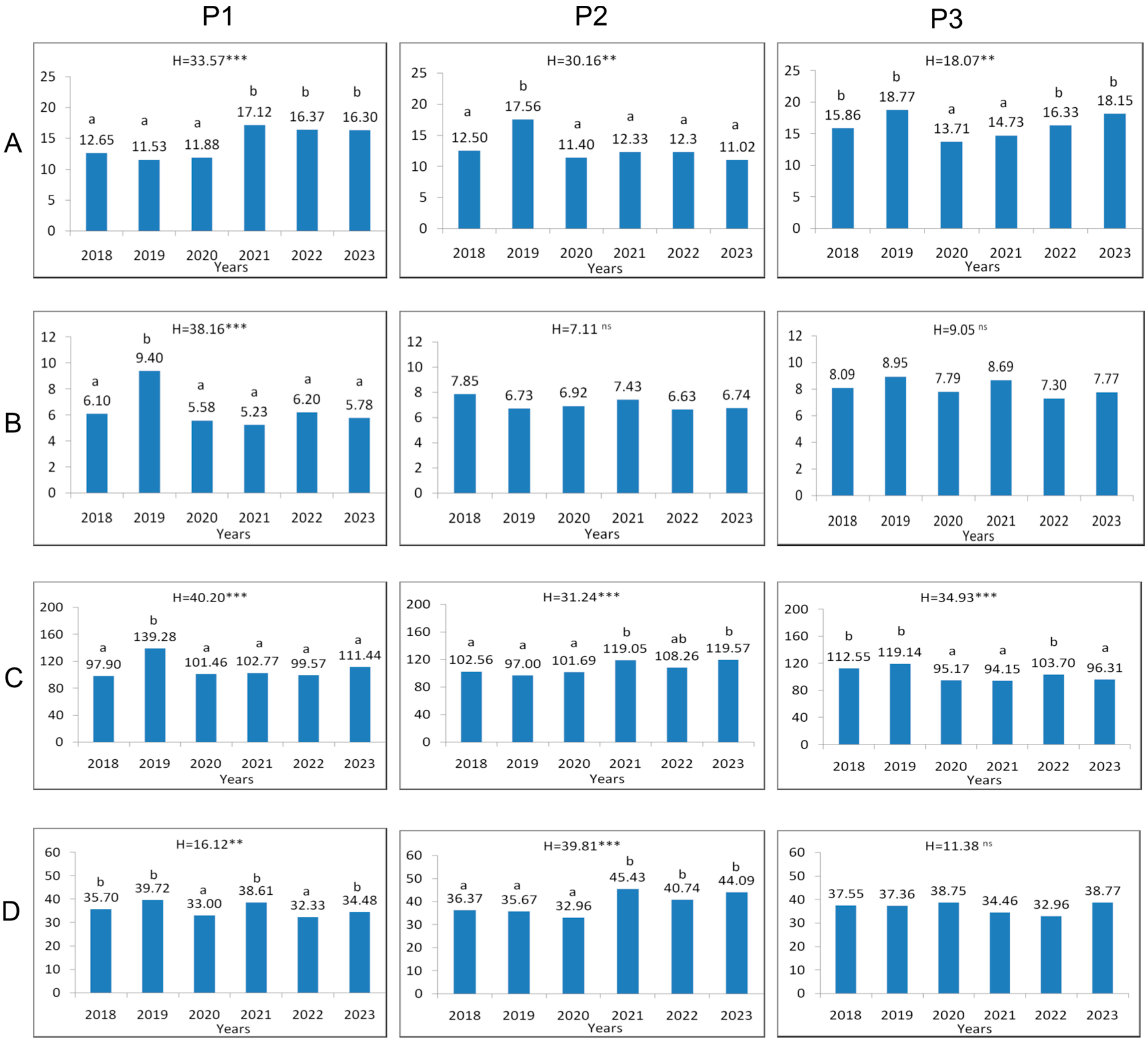

Figure S1. Mean height (A), number of leaves in the whorl (B), length (C) and width (D) of the longest leaf in the whorl of immature stems in population 1 located in Wolski Forest (P1), population 2 located in Mount Chełm (P2) and population 3 located in Hrabeński Forest (P3) in the years 2018–2023. The asterisks mean statistical significance level: *P≤0.05, **p<0.01, ***p<00.1 (The Kruskal–Wallis H test). The different letters below H values mean the significant differences among populations.

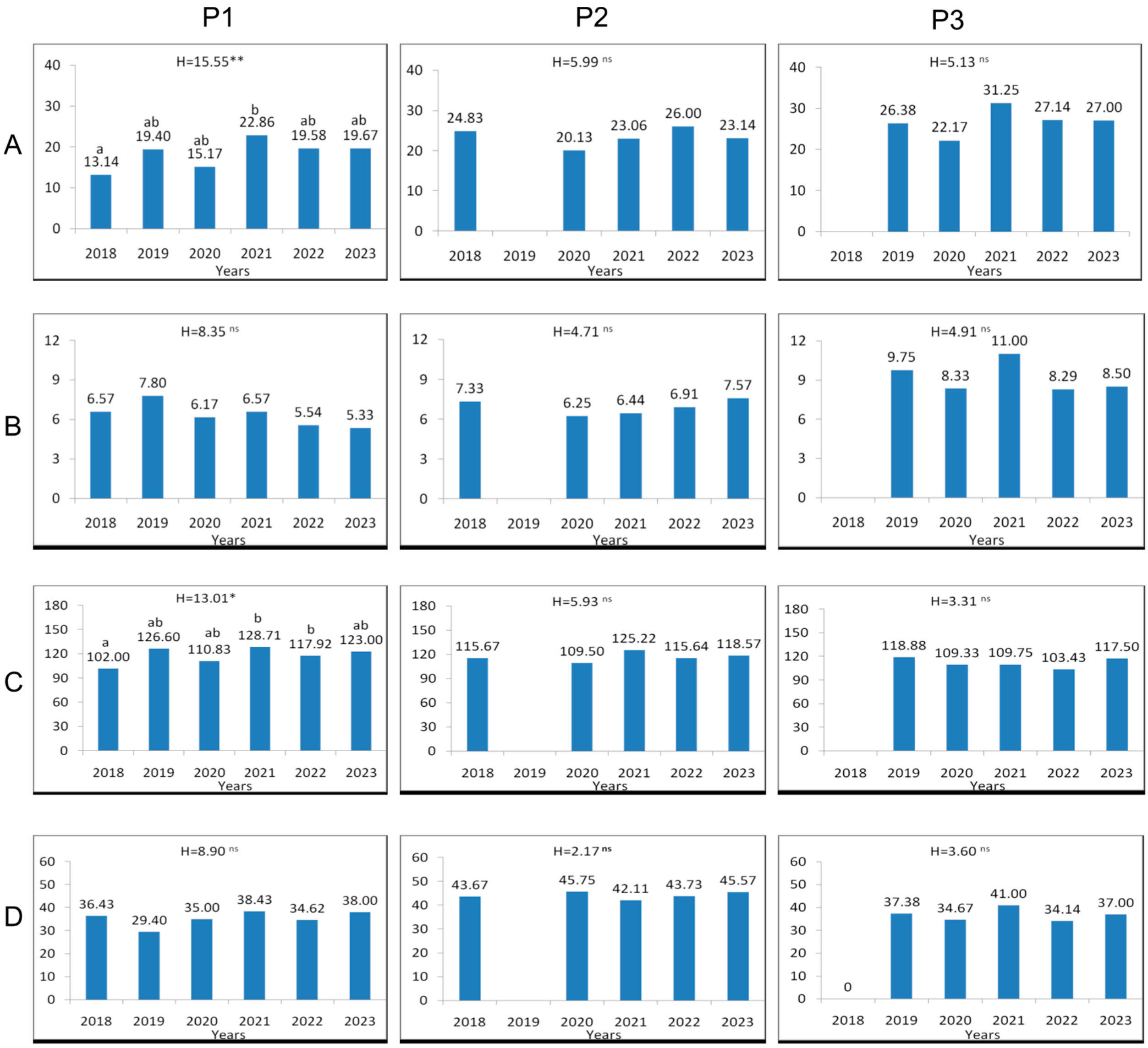

Figure S2. Mean height (A), number of leaves in the lower whorl (B), length (C) and width (D) of the longest leaf in the lower whorl of virginile stems in the observed populations. The explanations of abbreviations P1–P3 and description of statistical significance levels - as in Figure S1.

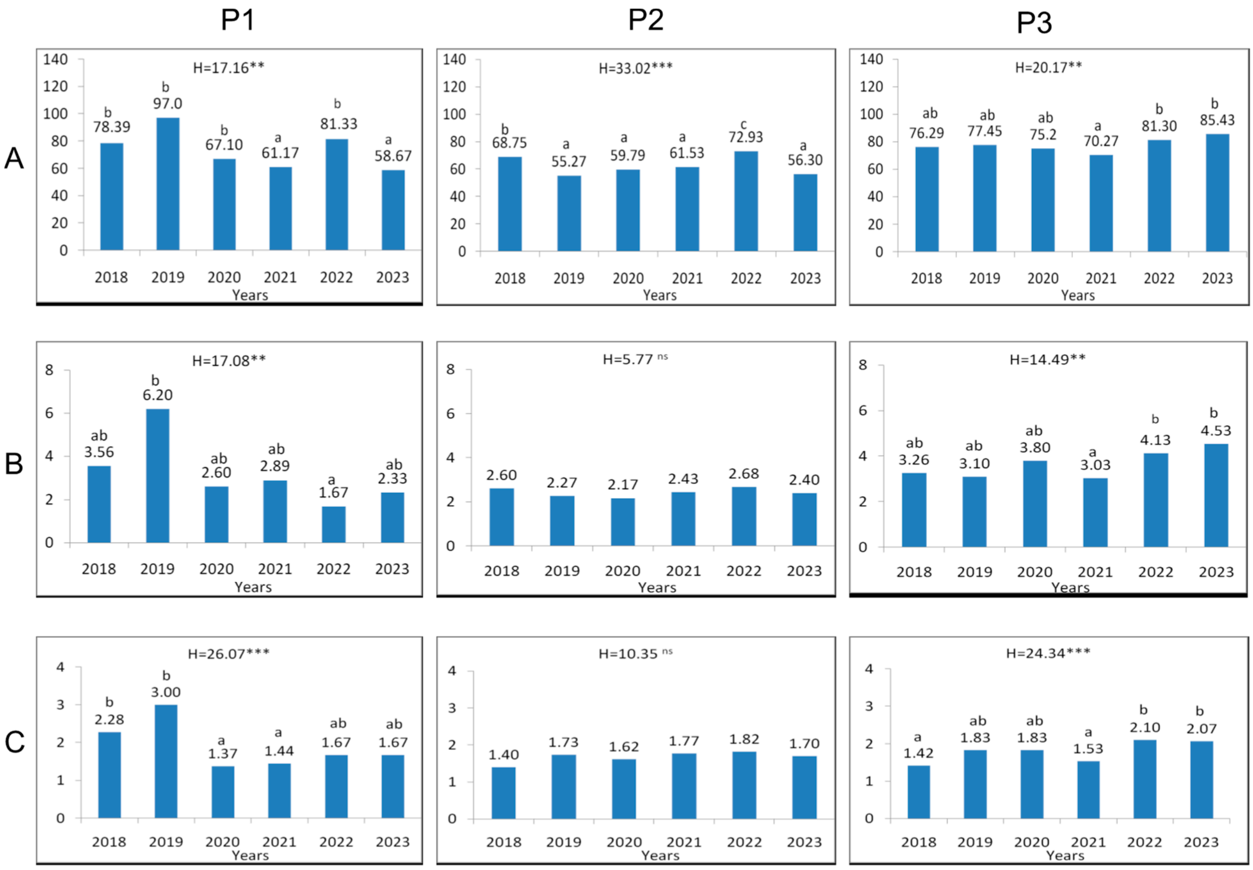

Figure S3. Mean height (A), number of flowers (B), and number of whorls (C) in generative stems in the ob-served populations. The explanations of abbreviations P1–P3 and description of statistical significance levels - as in Figure S1.

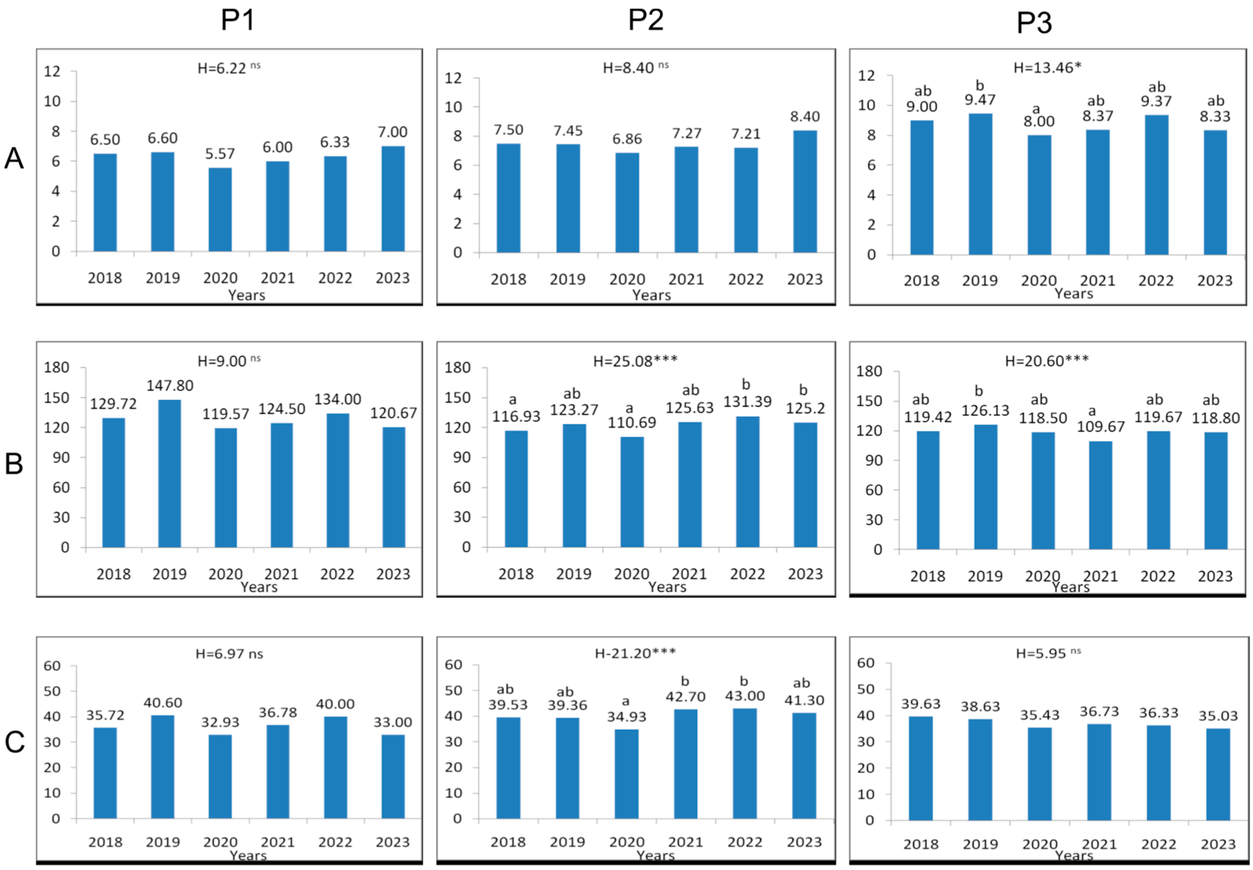

Figure S4. Mean number of leaves in the lowest whorl (A), length (B) and width (C) of the longest leaf in the lowest whorl in generative stems in the observed populations. The explanations of abbreviations P1–P3 and description of statistical significance levels - as in Figure S1.

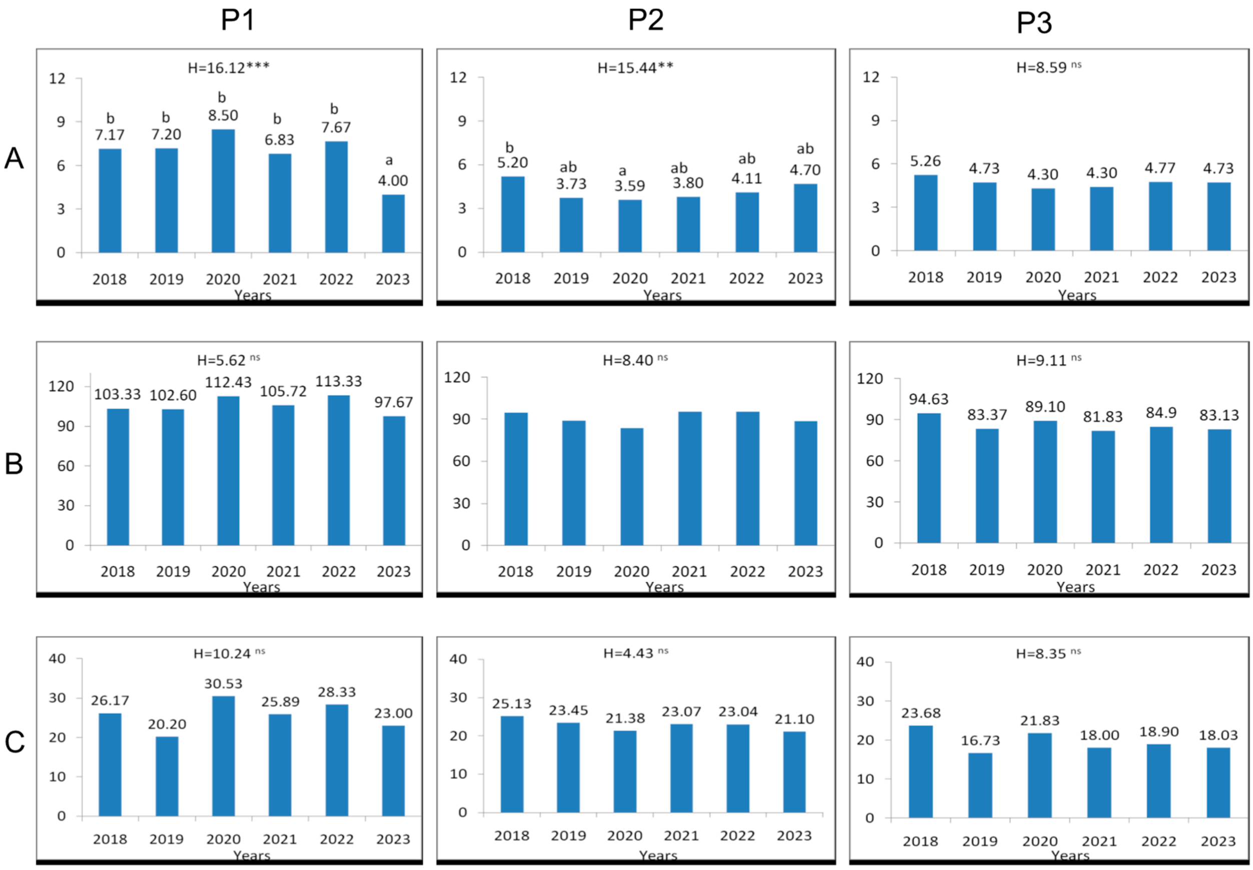

Figure S5. Mean number of spiral leaves (A), length (B) and width (B) of spiral leaf above the highest whorl in generative stems in the observed populations. The explanations of abbreviations P1–P3 and description of statistical significance levels - as in Figure S1.

Table S1. Values of the d-Cohen coefficient regarding the height, number of leaves in the whorl, and length and width of the longest leaf in the whorl of immature stems among pairs of study years in population 1 located in Wolski Forest, population 2 located on Mount Chełm, and population 3 located in Hrabeński Forest. Moderate (absolute value d>0.5) and substantial (absolute value d >0.8) differ-ences in the d-Cohen coefficient among the populations are highlighted in bold.

Population	Compared years	Height of stem [cm]	Number of leaves in the lower whorl	Length of leaves in the lower whorl [mm]	Width of leaves in the lower whorl [mm]
1	2018-2019	**-0.62**	**-0.89**	**-0.86**	-0.32
	2018-2020	0.14	0.18	-0.06	0.19
	2018-2021	0.02	0.25	-0.09	-0.13
	2018-2022	0.03	-0.04	-0.03	0.29
	2018-2023	0.09	0.04	-0.11	0.03
	2019-2020	**0.73**	**0.92**	**0.91**	0.46
	2019-2021	0.43	**0.86**	**0.92**	0.05
	2019-2022	**0.72**	**0.81**	**0.87**	**0.64**
	2019-2023	**0.85**	**0.84**	**0.73**	0.39
	2020-2021	-0.08	0.09	-0.03	-0.23
	2020-2022	-0.13	-0.20	0.03	0.05
	2020-2023	0.05	-0.06	-0.21	-0.10
	2021-2022	0.00	-0.26	0.06	0.29
	2021-2023	0.12	-0.13	-0.19	0.17
	2022-2023	0.21	0.13	-0.23	-0.17
2	2018-2019	0.14	0.30	0.15	0.05
	2018-2020	0.09	0.26	0.02	0.20
	2018-2021	**-0.51**	0.11	-0.45	-0.50
	2018-2022	-0.38	0.27	-0.14	-0.26
	2018-2023	-0.39	0.30	**-0.54**	-0.43
	2019-2020	-0.05	-0.05	-0.12	0.23
	2019-2021	**-0.74**	-0.18	**-0.60**	**-0.73**
	2019-2022	**-0.56**	0.02	-0.29	-0.43
	2019-2023	**-0.59**	-0.50	-0.40	**-0.60**
	2020-2021	**-0.68**	-0.14	-0.46	**-0.84**
	2020-2022	**-0.51**	0.07	-0.16	**-0.59**
	2020-2023	**-0.53**	0.05	-0.55	**-0.76**
	2021-2022	0.08	0.17	0.28	0.32
	2021-2023	0.09	0.18	-0.02	0.08
	2022-2023	0.01	-0.02	-0.34	-0.23
3	2018-2019	-0.43	-0.24	-0.22	0.02
	2018-2020	0.34	0.09	**0.57**	-0.10
	2018-2021	0.17	-0.14	**0.56**	0.22
	2018-2022	-0.02	0.08	0.07	0.10
	2018-2023	-0.34	0.09	**0.54**	-0.08
	2019-2020	**0.58**	0.35	**0.95**	-0.12
	2019-2021	**0.54**	0.06	**0.91**	0.23
	2019-2022	0.26	0.42	**0.51**	0.38
	2019-2023	0.07	0.33	**0.92**	-0.10
	2020-2021	-0.12	-0.23	0.04	0.31
	2020-2022	-0.28	0.11	-0.26	0.41
	2020-2023	-0.51	0.01	-0.04	0.00
	2021-2022	-0.17	0.31	-0.29	0.11
	2021-2023	-0.37	0.22	-0.08	-0.27
	2022-2023	-0.19	-0.12	0.20	-0.35

Table S2. Values of the d-Cohen coefficient regarding the height, number of leaves in the whorl, and length and width of the longest leaf in the whorl of virginile stems among pairs of study years in the observed populations. Population localities and description of d-Cohen coefficient values - as in Table S1.

Population	Compared years	Height of stem [cm]	Number of leaves in the lower whorl	Length of leaves in the lower whorl [mm]	Width of leaves in the lower whorl [mm]
1	2018-2019	**-0.70**	-0.35	**-0.92**	**0.69**
	2018-2020	-0.25	0.14	-0.34	0.09
	2018-2021	**-1.25**	0.00	**-1.08**	-0.12
	2018-2022	**-0.60**	0.41	**-0.57**	0.12
	2018-2023	-0.43	0.17	-0.18	-0.04
	2019-2020	**0.52**	**0.55**	**0.58**	**-0.75**
	2019-2021	-0.44	0.33	-0.08	**-1.08**
	2019-2022	-0.02	**0.88**	0.30	**-0.73**
	2019-2023	-0.04	**1.04**	0.13	**-2.43**
	2020-2021	**-1.08**	-0.13	**-0.71**	-0.24
	2020-2022	-0.44	0.32	-0.25	0.03
	2020-2023	**-0.78**	0.48	-0.44	-0.32
	2021-2022	0.34	0.39	0.40	0.28
	2021-2023	**0.58**	0.50	0.22	0.04
	2022-2023	-0.01	0.15	-0.17	-0.38
2	2018-2019	-	-	-	-
	2018-2020	**0.73**	**0.65**	0.35	-0.24
	2018-2021	0.22	0.41	-0.49	0.25
	2018-2022	-0.13	0.17	0.00	-0.01
	2018-2023	0.23	-0.09	-0.18	-0.23
	2019-2020	-	-	-	-
	2019-2021	-	-	-	-
	2019-2022	-	-	-	-
	2019-2023	-	-	-	-
	2020-2021	-0.47	-0.13	-0.85	0.33
	2020-2022	**-0.80**	-0.37	-0.26	0.14
	2020-2023	**-0.57**	**-0.70**	**-0.59**	0.01
	2021-2022	-0.33	-0.20	0.37	-0.13
	2021-2023	-0.01	-0.47	0.39	-0.33
	2022-2023	0.35	-0.25	-0.13	-0.13
3	2018-2019	-	-	-	-
	2018-2020	-	-	-	-
	2018-2021	-	-	-	-
	2018-2022	-	-	-	-
	2018-2023	-	-	-	-
	2019-2020	0.46	0.28	0.31	0.20
	2019-2021	-0.41	-0.25	0.38	-0.26
	2019-2022	-0.07	0.26	**0.56**	**0.24**
	2019-2023	-0.06	0.24	0.06	0.04
	2020-2021	**-0.90**	**-0.72**	-0.02	**-0.55**
	2020-2022	-0.41	0.01	0.23	0.04
	2020-2023	**-0.52**	-0.04	-0.35	-0.34
	2021-2022	0.34	**0.64**	0.24	**0.58**
	2021-2023	0.35	**0.63**	-0.49	**0.52**
	2022-2023	0.01	-0.06	**-0.52**	-0.37

Table S3. **The** values of d-Cohen coefficient regarding to height, number of flowers, number of whorls of generative stems among pairs of study years in observed populations. The population localities and description of d-Cohen coefficient values - as in Table S1.

Population	Compared years	Height of stem [cm]	Number of flowers	Number of whorls
1	2018-2019	**-0.58**	**-0.91**	-0.45
	2018-2020	0.25	0.25	**0.66**
	2018-2021	0.50	0.21	**0.59**
	2018-2022	-0.09	**0.79**	0.42
	2018-2023	0.22	0.30	0.21
	2019-2020	**0.84**	**1.15**	**1.36**
	2019-2021	**1.44**	**1.37**	**1.28**
	2019-2022	**0.63**	**2.71**	**1.04**
	2019-2023	**1.81**	**2.31**	**1.04**
	2020-2021	0.16	-0.09	-0.08
	2020-2022	-0.37	0.36	-0.28
	2020-2023	0.25	0.10	-0.28
	2021-2022	**-0.74**	**0.64**	-0.20
	2021-2023	0.11	0.29	-0.20
	2022-2023	**0.96**	**-0.58**	0.00
2	2018-2019	0.49	0.14	-0.34
	2018-2020	0.35	0.18	-0.22
	2018-2021	0.31	0.08	-0.37
	2018-2022	-0.17	-0.03	-0.47
	2018-2023	**0.51**	0.09	-0.31
	2019-2020	-0.17	0.05	0.11
	2019-2021	-0.26	-0.09	-0.04
	2019-2022	**-0.69**	-0.22	-0.11
	2019-2023	-0.44	-0.48	**-0.51**
	2020-2021	-0.08	-0.15	-0.15
	2020-2022	**-0.56**	-0.28	-0.23
	2020-2023	0.15	-0.13	-0.08
	2021-2022	**-0.54**	-0.14	-0.06
	2021-2023	0.25	0.02	0.07
	2022-2023	**0.74**	0.16	0.14
3	2018-2019	-0.05	0.07	-0.47
	2018-2020	0.05	-0.18	-0.43
	2018-2021	0.27	0.09	-0.11
	2018-2022	-0.06	-0.19	-0.33
	2018-2023	-0.33	-0.35	**-0.59**
	2019-2020	0.09	-0.26	0.00
	2019-2021	0.31	0.03	0.34
	2019-2022	-0.15	-0.42	-0.26
	2019-2023	-0.28	-0.43	-0.24
	2020-2021	0.21	0.27	0.31
	2020-2022	-0.24	-0.13	-0.23
	2020-2023	-0.35	-0.19	-0.22
	2021-2022	-0.43	-0.43	-0.48
	2021-2023	**-0.54**	-0.45	-0.49
	2022-2023	-0.15	-0.14	0.03

Table S4. Values of the d-Cohen coefficient regarding the number of leaves in the lowest whorl, length and width of the longest leaf in the lowest whorl, number of spiral leaves, and length and width of the spiral leaf above the highest whorl of generative stems among pairs of study years in the observed populations. Population localities and description of d-Cohen coefficient values - as in Table S1.

Population	Compared years	Number of leaves in the lowest whorl	Length of the longest leaf in the lowest whorl [mm]	Width of the longest leaf in the lowest whorl [mm]	Number of spiral leaves	Length of spiral leaf above the highest whorl [mm]	Width of the spiral leaf above the highest whorl [mm]
1	2018-2019	-0.02	-0.43	-0.29	-0.01	0.03	**0.68**
	2018-2020	0.30	0.31	0.20	-0.32	-0.31	-0.30
	2018-2021	0.16	0.14	-0.08	0.06	-0.06	0.02
	2018-2022	0.07	-0.11	-0.28	-0.18	**-0.51**	-0.22
	2018-2023	-0.08	0.06	0.05	0.39	0.04	0.09
	2019-2020	0.25	**0.77**	**0.56**	-0.27	-0.38	**-1.10**
	2019-2021	0.15	**0.55**	0.24	0.06	-0.09	-0.44
	2019-2022	0.08	0.33	0.03	-0.14	**-0.64**	**-1.74**
	2019-2023	-0.15	**0.51**	0.31	**0.85**	0.13	-0.25
	2020-2021	-0.15	-0.15	-0.29	0.30	0.16	0.25
	2020-2022	-0.38	-0.44	-0.46	0.32	-0.04	0.21
	2020-2023	**-1.00**	-0.03	0.00	**1.48**	0.32	0.44
	2021-2022	-0.17	-0.25	-0.22	-0.21	-0.23	-0.17
	2021-2023	**-0.73**	0.08	0.18	**0.63**	0.15	0.14
	2022-2023	-1.15	0.27	0.30	**2.32**	0.43	0.43
2	2018-2019	0.01	-0.22	0.01	0.47	0.16	0.10
	2018-2020	0.19	0.21	0.31	0.46	0.29	0.22
	2018-2021	0.07	-0.33	-0.28	0.42	-0.02	0.15
	2018-2022	0.08	-0.47	-0.28	0.31	-0.02	0.14
	2018-2023	-0.27	-0.34	-0.16	0.15	0.16	0.26
	2019-2020	0.15	0.38	0.31	0.06	0.14	0.11
	2019-2021	0.05	-0.08	-0.30	-0.03	-0.20	0.03
	2019-2022	0.06	-0.24	-0.30	-0.15	-0.18	0.03
	2019-2023	-0.42	-0.43	-0.48	**-0.56**	**-0.55**	-0.46
	2020-2021	-0.13	-0.49	**-0.54**	-0.08	-0.33	-0.11
	2020-2022	-0.10	**-0.59**	**-0.52**	-0.18	-0.31	-0.10
	2020-2023	-0.46	-0.50	-0.46	-0.39	-0.11	0.02
	2021-2022	0.02	-0.18	-0.02	-0.11	0.00	0.00
	2021-2023	-0.34	0.02	0.13	-0.33	0.19	0.15
	2022-2023	-0.33	0.20	0.14	-0.20	0.18	0.14
3	2018-2019	-0.10	-0.22	0.08	0.16	0.33	**0.52**
	2018-2020	0.31	0.03	0.27	0.34	0.19	0.07
	2018-2021	0.18	0.29	0.22	0.24	0.37	0.34
	2018-2022	-0.03	0.00	0.07	0.07	0.09	0.17
	2018-2023	0.20	0.02	0.39	0.17	0.42	0.44
	2019-2020	0.35	0.35	0.20	0.13	-0.17	-0.22
	2019-2021	0.25	**0.61**	0.13	0.08	0.04	-0.08
	2019-2022	0.02	0.23	0.17	-0.01	-0.05	-0.20
	2019-2023	0.26	0.27	0.29	0.00	0.01	-0.12
	2020-2021	-0.12	0.35	-0.08	-0.04	0.21	0.15
	2020-2022	-0.25	-0.04	-0.07	-0.11	0.12	0.21
	2020-2023	-0.11	-0.01	0.03	-0.15	0.21	0.17
	2021-2022	-0.18	-0.32	0.03	-0.08	-0.09	-0.07
	2021-2023	0.01	-0.30	0.13	-0.09	-0.04	0.00
	2022-2023	0.19	0.03	0.11	0.01	0.06	0.07
